# CB1R-stabilized NLRP3 inflammasome drives antipsychotics cardiotoxicity

**DOI:** 10.1038/s41392-022-01018-7

**Published:** 2022-06-24

**Authors:** Liliang Li, Pan Gao, Xinru Tang, Zheng Liu, Mengying Cao, Ruoyu Luo, Xiaoqing Li, Jing Wang, Xinyi Lin, Chao Peng, Zhihong Li, Jianhua Zhang, Xian Zhang, Zhonglian Cao, Yunzeng Zou, Li Jin

**Affiliations:** 1 Department of Forensic Medicine, School of Basic Medical Sciences, Fudan University, Shanghai, 200032 China; 2 State Key Laboratory of Genetic Engineering, Collaborative Innovation Center for Genetics and Development, School of Life Sciences & Human Phenome Institute, Fudan University, Shanghai, 200438 China; 3 Shanghai Institute of Cardiovascular Diseases, Zhongshan Hospital and Institutes of Biomedical Sciences, Fudan University, Shanghai, 200032 China; 4 National Facility for Protein Science in Shanghai, Zhangjiang Lab, Shanghai Advanced Research Institute, Chinese Academy of Science, Shanghai, 201210 China; 5 Academy of Forensic Science, Ministry of Justice, and Shanghai Key Laboratory of Forensic Medicine, Shanghai, 200063 China; 6 Department of Cardiology, Kunshan Hospital of Integrated Traditional Chinese and Western Medicine, Kunshan, Jiangsu 215301 China; 7 School of Pharmacy, Fudan University, Shanghai, 201203 China; 8 Shanghai Medical College, Fudan University, Shanghai, 200032 China

**Keywords:** Cardiology, Drug safety

## Abstract

Long-term use of antipsychotics is a common cause of myocardial injury and even sudden cardiac deaths that often lead to drug withdrawn or discontinuation. Mechanisms underlying antipsychotics cardiotoxicity remain largely unknown. Herein we performed RNA sequencing and found that NLRP3 inflammasome-mediated pyroptosis contributed predominantly to multiple antipsychotics cardiotoxicity. Pyroptosis-based small-molecule compound screen identified cannabinoid receptor 1 (CB1R) as an upstream regulator of the NLRP3 inflammasome. Mechanistically, antipsychotics competitively bond to the CB1R and led to CB1R translocation to the cytoplasm, where CB1R directly interacted with NLRP3 inflammasome *via* amino acid residues 177–209, rendering stabilization of the inflammasome. Knockout of *Cb1r* significantly alleviated antipsychotic-induced cardiomyocyte pyroptosis and cardiotoxicity. Multi-organ-based investigation revealed no additional toxicity of newer CB1R antagonists. In authentic human cases, the expression of CB1R and NLRP3 inflammasome positively correlated with antipsychotics-induced cardiotoxicity. These results suggest that CB1R is a potent regulator of the NLRP3 inflammsome-mediated pyroptosis and small-molecule inhibitors targeting the CB1R/NLRP3 signaling represent attractive approaches to rescue cardiac side effects of antipsychotics.

## Introduction

Antipsychotic (AP) drugs are common agents for treating mental disorders such as schizophrenia, bipolar disorder, and major depressive disorder. Overall AP prescription increased 3.8-fold, while second-generation AP (SGA) prescriptions increased 18.1-fold during a 16-year follow-up.^1^ The increased use of APs has led to public concern on the cardiac adverse effect, termed as cardiotoxicity, which is an uncommon but deadly effect, in addition to metabolic abnormalities.^2,3^ It has been estimated that long-term use of first-generation AP (FGA) and SGA drugs showed higher rates of sudden cardiac death than did nonusers of AP drugs, with adjusted incidence-rate ratios of 1.99 and 2.26, respectively.^4^

AP drug use associates with inflammatory lesions and cardiomyopathy.^5,6^ Multiple AP drugs have been reported to impair cardiac structure and function, causing diffuse fibro-inflammatory myocardial process in patients.^7^ Specifically, AP drugs could reduce myocyte viability,^8^ potentially leading to cardiac fibrosis,^9^ a condition that predisposes to arrhythmogenic sudden cardiac death.^10^ In the clinic, multiple AP drugs have been documented to block repolarizing potassium currents and prolong QT interval,^11,12^ one causal mechanism for the ventricular tachyarrhythmias that often lead to sudden cardiac death.^13^ This was consistent with an autopsy-based retrospective study that tabulated clozapine (Clz), chlorpromazine (CPZ), olanzapine (Olz), and quetiapine (Que) as the most common AP drugs associate with sudden deaths.^14^ Indeed, the severe cardiotoxicity of Clz has drawn attention of the Food and Drug Administration, leading to the cautious use of Clz in clinic. Despite extensive clinical concern on the AP drug safety, the rescue strategy has remained largely unchanged for years. When situated for AP drugs-induced dysrhythmia, administrative offices or clinicians usually withdraw or discontinue the offending drugs, a passive action that unfortunately leads to economic waste of on-the-market drugs. Therefore, in-depth investigation of APs cardiotoxicity with systemic evidence remains urgently needed.

Since the cardiac structure impairment fundamentally originates from excessive cell death by toxic insults, profiling of molecular alterations would shed lights into drug-toxicity prevention.^15^ The present study, therefore, started with systemic investigation of the molecular and cellular details in response to chronic APs uses. We hypothesized that cell death pathways driven by these altered molecules would play critical roles in AP drug cardiotoxicity, and a cell death-based small-molecule compound screen would help to identify cardioprotectants against antipsychotics use.

## Results

### AP drugs induced cardiotoxicity at early stage

**Fig. 1:**
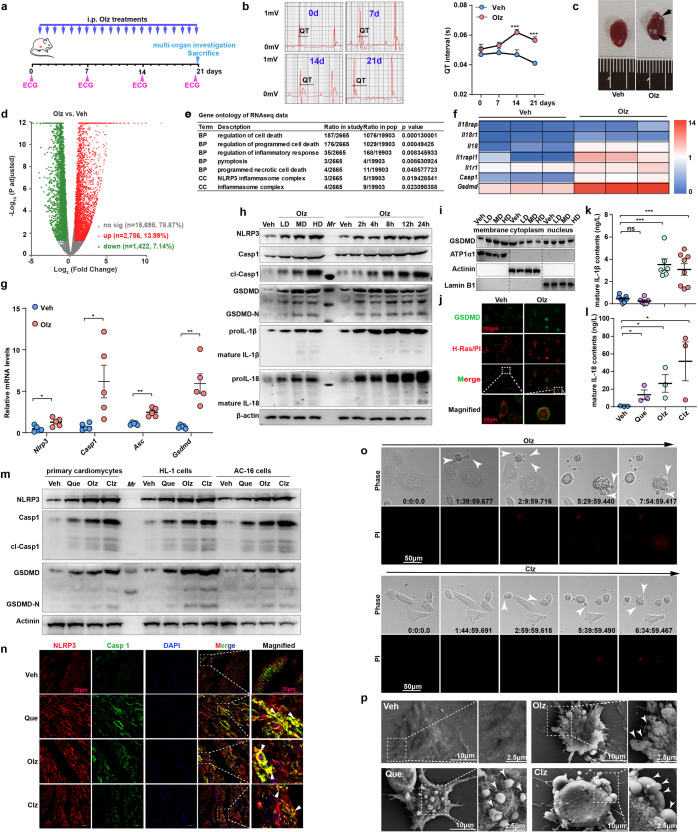
Multiple AP drugs activated the NLRP3 inflammasome and myocyte pyroptotic death. **a** Schematic illustration of the study design. **b** Electrocardiography (ECG) monitoring of mice electrical activity in 0, 7, 14, and 21 days (*n* = 6 mice/group). **c** Gross images of vehicle (Veh, PBS) and Olz (5 mg/kg)-treated (21 days) mouse hearts. Black arrow indicated exudative patches over epicardium. **d**–**f** RNA sequencing of total RNAs from Veh (PBS) and Olz-treated mouse hearts (21 days). **g** qRT-PCR validation of gene expression including *Nlrp3*, *Casp1*, *Asc*, and *Gsdmd* in mouse hearts (*n* = 5 mice/group). **h** Western blot analysis of the NLRP3 inflammasome proteins in mouse HL-1 cells under Veh (PBS), low dose (LD, 1 μM), medium dose (MD, 4 μM), and high dose (HD, 16 μM) of Olz treatments for 24 h, or under constant Olz treatments (4 μM) for different hours. **i** Subcellular components were isolated from mouse HL-1 cells at indicated treatments for 24 h, and western blot analysis of GSDMD was performed. **j** Immunofluorescence assay analysis of GSDMD (green signal) localization. H-Ras (red signal) is a membrane marker, and cell membrane integrity was monitored by propidium iodide (PI, 3 μM) uptake. Scale bar = 100 μm. **k**, **l** Enzyme-linked immunosorbent assay (ELISA) analysis of mature IL-1β and IL-18 contents in supernatants of mouse HL-1 cells under Veh (PBS), quetiapine (Que, 4 μM), Olz (4 μM), and clozapine (Clz, 20 μM) treatments for 24 h. **m** Western blot analysis of the NLRP3 inflammasome and pyroptosis in primary mouse cardiomyocytes, mouse HL-1 cell line, and human AC-16 cells under Veh (PBS), Clz (20 μM), Olz (4 μM), and Que (4 μM) treatments for 24 h. **n** Immunofluorescence analysis of NLRP3 (red) and Casp1 (green) in mouse hearts receiving indicated treatments. Arrowheads indicated merged NLRP3 and Casp1 signals (yellow). Scale bar = 20 μm. **o** Time-lapse microscopy of Olz (4 μM) and Clz (20 μM)-treated rat H9c2 myocytes. Cell morphology was visualized by wide-field light microscopy (upper panel) and cell membrane integrity was monitored by PI uptake (lower panel). Arrowheads indicated pyroptotic cells with protrusions. Scale bar = 50 μm. Time duration = h: min: s. ms. **p** Scanning electron microscopy of Veh (PBS), Olz (4 μM), Clz (20 μM), and Que (4 μM)-treated rat H9c2 myocytes. Arrowheads indicated bubbling of pyroptotic cells. All quantification represents the mean and SEM of independent experiments. Two-way ANOVA was used for analysis in (**b**). The Student’s *t*-test was used for analysis in (**g**, **k**, **l**). ns, no significance. *p < 0.05; **p < 0.01; ***p < 0.001 as indicated

In a 21-day treatment period, we selected Olz, one of the first-line clinically used SGAs, as a representative drug to treat mice on a daily basis, and multi-organ-based toxicity assessment was then performed (Fig. 1a). Since all mice were monitored at a heart rate of approximately 400 bpm, we used QT interval without correction in the electrocardiographic recording. It showed that QT interval was significantly prolonged by approximate 30% since day 14, and the amplitude of J-wave, an indicator of local ischemia, also tended to be elevated by Olz (Fig. 1b). Olz-treated hearts were grossly enlarged and presented with exudative patches (Fig. 1c, black arrow). AP drugs are extensively documented to induce metabolic abnormalities in clinical observations.^3^ In the 21-day period, we found that the random blood glucose levels were comparable between groups, and fast blood glucose levels were modestly increased after Olz treatments (Supplementary Fig. 1a–c). Chronic APs treatments did not induce significant body weight gains, serum TG and TC elevations, and histological analysis did not reveal remarkable alterations of major metabolic organs within 21-day period (Supplementary Fig. 1d–l). LC-MS/MS studies showed that the serum Olz and Clz concentrations fluctuated among days (Supplementary Fig. 2), and were generally within therapeutic ranges after mapping to the concentration references;^16^ However, the Olz and Clz concentrations in the heart were significantly elevated since day 14 and its average level increased by up to 3-fold and 22-fold on day 21, respectively, as compared with day 7 while these two APs and their major metabolites remained relatively stable in mouse liver and kidney, albeit some fluctuation among the periods (Supplementary Fig. 2). These observations suggested that AP drugs accumulated in the heart and exclusively induced cardiac toxic effects before the development of systemic glycolipid metabolism disorder or other organ dysfunctions.

### Multiple AP drugs activated NLRP3 inflammasome and induced myocyte pyroptosis

We then performed RNA sequencing of the Olz-treated hearts. A total of 19,903 genes were identified, among which 2786 genes (13.99%) were significantly upregulated and 1422 genes (7.14%) were significantly downregulated (Fig. 1d). Gene Ontology (GO) analysis revealed that multiple programmed cell death pathways, particularly inflammasome-mediated pyroptosis, were significantly enriched (Fig. 1e). Upregulated pyroptosis genes were illustrated in Fig. 1f and verified by qRT-PCR analysis (Fig. 1g). Western blot analysis also revealed that Olz enhanced the protein levels of NLRP3 inflammasome and pro-inflammatory factors in a dose- and time-dependent manner (Fig. 1h). The pyroptosis executor GSDMD was exported from cytoplasm to membrane by higher doses of Olz treatments (Fig. 1i). GSDMD translocation was further confirmed by its co-localization with membrane-resided H-Ras protein, and was observed to accompany with cell uptake of propidium iodide (PI) (Fig. 1j), a dye that penetrates into cells when membrane integrity is disrupted. Following the membrane-residency of GSDMD, three commonly prescribed SGAs, Que, Olz, and Clz, remarkably promoted the secretion of mature IL-1β (Fig. 1k) and IL-18 (Fig. 1l) in mouse HL-1 myocytes. In addition, the three AP drugs also induced pyroptosis protein expression in primary mouse cardiomyocytes and in a human cardiomyocyte (AC-16 cells) (Fig. 1m). We further used the Olz-treated mouse hearts, together with previously Clz-treated^17^ and Que-treated^18^ mouse hearts, for in vivo observations. Immunofluorescence staining showed that these AP drug-treated mouse hearts consistently exhibited higher fluorescence intensity of NLRP3 (red) and Casp1 (green) and displayed well-merged signals (yellow) that indicated the NLRP3 inflammasome specks (Fig. 1n, white arrowheads).

To obtain morphological evidence, using time-lapse microscopy, we found that Olz-treated myocytes began to detach about 1.5 h after exposure and displayed multiple bubble-like protrusions (indicated by arrowheads), and progressed to pyroptotic cell death in conjunction with PI uptake about 5.5 h after drug exposure (Fig. 1o and Supplementary Video 1). Clz-treated myocytes exhibited similar phenomenon (Fig. 1o and Supplementary Video 2). Scanning electron microscopy further confirmed the activation of pyroptosis upon APs treatment (Fig. 1p). To further confirm involvement of the NLRP3 inflammasome in AP drug-induced pyroptosis, we synthesized a pool of shRNAs targeting each of the pyroptosis genes and found that Olz failed to induce intense GSDMD membrane location when either *Nlpr3*, *Casp1*, or *Gsdmd* was depleted (Supplementary Fig. 3a–d). This was further verified when a specific NLRP3 inhibitor MCC950 obviously dampened the Olz-induced pyroptotic protrusions (indicated by white arrows) and PI uptake (Supplementary Fig. 3e).

To assess the specificity of pyroptosis in AP drug cytotoxicity, we detected the expression of other cell death markers. We found that apoptosis and autophagy tended to be mildly enhanced and necroptosis remained barely activated by Olz treatments (Supplementary Fig. 4a, b). Olz-treated myocytes showed evidence of osmotic lysis with organelle swelling, membrane protrusions (indicated by white arrowhead) and vesicles containing electron-dense contents (black arrowheads) as well as remarkable autolysosome (white arrows) (Supplementary Fig. 4c). We then utilized small-molecule inhibitors targeting each of these cell-death types. It showed that autophagy inhibitors (3-Methyladenine and wortamanin), apoptosis inhibitors, or a necroptosis inhibitor (necrostatin-1) failed to rescue Olz-induced cardiac injuries, whereas inhibitors of the NLRP3 inflammasome (MCC950 and VX-765) succeeded to rescue Olz-induced HL-1 and H9c2 cell damage (Supplementary Fig. 4d, e). These observations suggested multiple AP drugs prominently induced myocyte pyroptotic death.

### Genetic knockout or pharmacologic inhibition of NLRP3 inflammasome abrogated AP drug-induced myocardial injuries

**Fig. 2:**
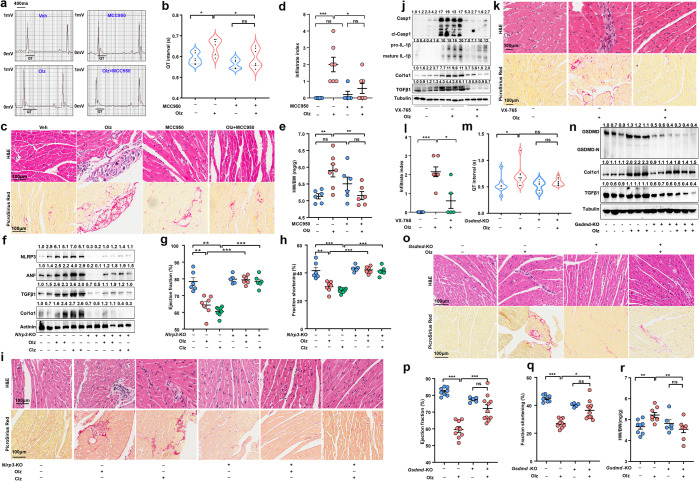
Genetic knockout or pharmacologic inhibition of cell pyroptosis significantly alleviated antipsychotic-induced myocardial injuries. **a**–**e** Effects of MCC950 (20 mg/kg), a specific NLRP3 inhibitor, on Olz cardiotoxicity (*n* = 5–7 mice/group). **f**–**i** Effects of genetic knockout of *Nlrp3* (*Nlrp3*-KO) on AP drugs-induced activation of pyroptosis and cardiotoxicity were assessed (*n* = 5–8 mice/group). The values above each protein blot (**f**) represent the ratio of protein intensity to actinin. **j**, **k** Effects of VX-765 (50 mg/kg), a specific Casp1 inhibitor, on Olz cardiotoxicity (*n* = 5 mice/group). The values above each protein blot (**j**) represent the ratio of protein intensity to tubulin. **l**–**r** Effects of genetic knockout of *Gsdmd* (*Gsdmd*-KO*)* on Olz cardiotoxicity (*n* = 6–9 mice/group). Scale bar = 100μm. The values above each protein blot (**n**) represent the ratio of protein intensity to tubulin. All quantification represents the mean and SEM of each group. Two-way ANOVA was used for all analyses. ns, no significance. *p < 0.05; **p < 0.01; ***p < 0.001 as indicated

To assess whether NLRP3 inflammasome-mediated pyroptosis had functional association with AP drug-induced cardiotoxic effects, we next retarded cell pyroptosis using genetic and pharmacologic approaches. It was observed that pretreatments with the NLRP3 inhibitor MCC950 restored Olz-induced QT interval prolongation (Fig. 2a, b) and limited the inflammatory infiltrates and fibrosis (Fig. 2c, d). As a consequence of the NLRP3 inhibition, the ratio of HW to BW significantly decreased to baseline (Fig. 2e). Similarly, knockout of *Nlrp3* (*Nlrp3*-KO) significantly alleviated the Olz or Clz-induced cardiac expression of pro-fibrotic factors (TGFβ1 and Col1α1) and ANF (Fig. 2f), restored AP drugs-induced left-ventricle dysfunction (Fig. 2g, h), and prevented AP drugs-induced inflammatory infiltrates and fibrotic tissue accumulation (Fig. 2i). The use of a Casp1 inhibitor VX-765 also limited the Olz-induced inflammatory reactions and fibrosis (Fig. 2j–l) in another mouse model.

We further genetically depleted the pyroptosis effector gene *Gsdmd (Gsdmd*-KO). It showed that in great contrast to wild-type (WT) mice, the *Gsdmd*-KO mice showed normalized QT interval (Fig. 2m) and presented lower cardiac expression of pro-fibrotic factors (TGFβ1 and Col1α1) even under the Olz stimuli (Fig. 2n). Histologically, the *Gsdmd*-KO mice were observed to be resistant to inflammatory infiltrates and fibrosis (Fig. 2o), and presented normalized left-ventricle functions (Fig. 2p, q) even under Olz treatments. The HW of *Gsdmd*-KO mice under Olz treatments was significantly lower than that of WT mice receiving Olz treatments (Fig. 2r). These results suggested that the NLRP3 inflammasome-mediated pyroptosis underlined AP drugs cardiotoxicity.

### Cannabinoid receptor 1 (CB1R) was a direct target of AP drugs and critically regulated the NLRP3 inflammasome

**Fig. 3:**
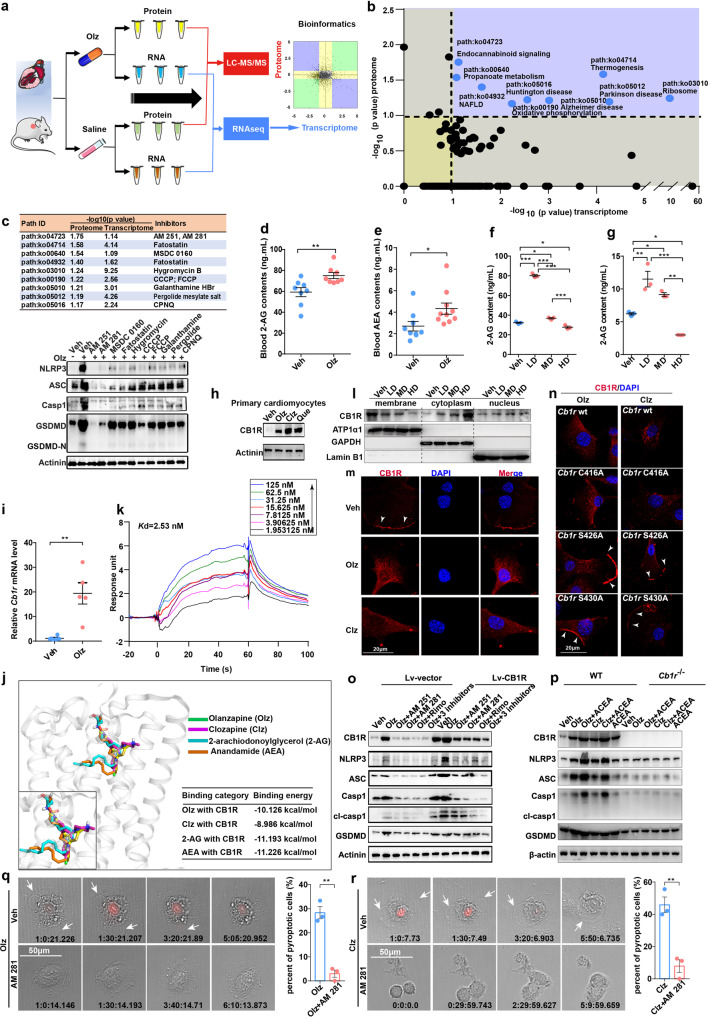
Pyroptosis-based screen identified cannabinoid receptor 1 (CB1R) as a critical regulator of cell pyroptosis. **a** Dual omics-based screen workflow. **b** Analysis of overlapped pathways by the transcriptomic and proteomic analyses. The overlapped pathways were highlighted in the right upper quadrant with the pathway ID and full names annotated. **c** Small-molecule compounds against the overlapped pathways were designed (upper panel) and added to the HL-1 myocytes for 24 h at a final dose of 1 μM. Western blot analysis was performed to assess the effects of pathway inhibition on the Olz-activated pyroptosis (lower panel). **d**, **e** LC-MS/MS detection of serum major endocannabinoids 2-arachiodonoylglycerol (2-AG) and anandamide (AEA) (*n* = 8–10/group) in Veh (PBS) or Olz (5 mg/kg)-treated mice. LC-MS/MS detection of cellular 2-AG levels in H9c2 cells (**f**) and HL-1 cells (**g**) receiving Veh (PBS), low dose (LD, 1 μM), medium dose (MD, 4 μM), and high dose (HD, 16 μM) of Olz from three independent assays. **h** Western blot analysis of CB1R in primary cardiomyocytes. **i** qRT-PCR analysis of *Cb1r* mRNA in Veh (PBS) or Olz (5 mg/kg)-treated mouse hearts (n = 5/group). **j** Molecular docking of ligand binding with human CB1R (hCB1R). The receptor is shown in gray cartoon representation. Ligands are shown in stick representation with indicated colors. The inset shows magnified view of ligand binding with CB1R. The binding energies were tabulated. **k** Surface plasmon resonance (SPR) measurements illustrating binding of Olz to the hCB1R. **l** Western blot detection of CB1R subcellular localization in response to Veh (PBS), LD (1 μM), MD (4 μM), and HD of Olz (16 μM) treatments of mouse HL-1 myocytes. **m** Subcellular localization of CB1R in response to clozapine (Clz, 20 μM) or Olz (4 μM) treatments of mouse HL-1 cells for 24 h. White arrowheads indicate membrane location. Scale bar = 20 μM. **n** Wild type (WT) *Cb1r* plasmid, *Cb1r* with C416A (CB1R-C416A), S426A (CB1R-S426A), or S430A (CB1R-S430A) mutants were individually transfected into *Cb1r-*knockout HL-1 myocytes and were then treated with Olz (4 μM) or Clz (20 μM) for 24 h. Images were taken under confocal immunofluorescence microscope. White arrowheads indicate CB1R membrane localization. Scale bar = 20 μM. **o**, **p** Cardiac HL-1 cells with stable overexpression of *Cb1r* (Lv-CB1R) and its control cells (Lv-vector), or CRISPR-mediated *Cb1r* knockout (*Cb1r*^*−/−*^) cells and the WT (*Cb1r*^+/+^) cells were cultured with indicated treatments for 24 h. Final doses of drugs were Olz (4 μM), Clz (20 μM), ACEA (2.7 μM), and each CB1R antagonist (1 μM). Western blot analysis was performed to detect pyroptosis proteins. **q**, **r** Rat H9c2 cells were pretreated with PBS or AM 281 (1 μM) for 1 h. The cells were then subject to Olz (4 μM) or Clz (20 μM) treatments in medium containing PI dye (30 μM). Time duration = h: min: s: ms. The percent of pyroptosis cells was quantified from three independent assays. Scale bar = 50 μm. All quantification represents the mean and SEM of each group. Students’ *t-*test was used for analysis in (**d**, **e**, **I**, **q**, **r**) and one-way ANOVA with Bonferroni post hoc test was used for analysis in (**f**, **g**). *p < 0.05; **p < 0.01; ***p < 0.001 as indicated

To uncover the primary mechanisms accounting for AP drug-activated pyroptosis, we further performed both transcriptome and proteome analyses, and analyzed the overlapped pathways by the dual-omics (Fig. 3a). A total of nine pathway hits were screened (Fig. 3b). Small-molecule compounds targeting each of these pathways were then designed (Fig. 3c) and a pyroptosis-based screen strategy was conducted. It turned out that many inhibitors could blunt Olz-induced expression of the NLRP3 inflammasome, among which inhibitors of endocannabinoid system (AM 251 and AM 281) showed the most prominent inhibition efficiency (Fig. 3c). To confirm the involvement of the endocannabinoid system, we developed a sensitive LC-MS/MS method^19^ and detected the major endocannabinoids, 2-arachiodonoylglycerol (2-AG) and arachidonylethanolamine (or anandamide, AEA). It turned out that the serum contents of both 2-AG and AEA were significantly increased after 21 days’ Olz treatments (Fig. 3d, e). In both rat H9c2 (Fig. 3f) and mouse HL-1 myocytes (Fig. 3g), the cellular contents of 2-AG were detected to be increased by low dose (LD) and significantly decreased by high dose (HD) of Olz, mirroring the passive changes of endogenous ligands by Olz doses.

Since *Cb1r* was the only receptor that was identified from the endocannabinoid system by the transcriptome, we then asked whether CB1R was directly affected by AP drug treatments. The expression of CB1R was detected to be promoted in Olz, Clz, or Que-exposed primary mouse cardiomyocytes (Fig. 3h), and its mRNA level was significantly increased in Olz but not Veh (PBS)-treated mouse hearts (Fig. 3i). Furthermore, molecular docking analysis showed that both the AP drugs (Olz and Clz) and the major endocannabinoids (2-AG and AEA) bound individually to the CB1R structural fold at indicated residues (Supplementary Fig. 5a–d). Importantly, Olz, Clz, 2-AG, and AEA shared highly overlapped docking architecture within the binding pocket of CB1R (Fig. 3j). Surface plasmon resonance analysis confirmed that Olz directly bond to the purified hCB1R with a *K*d value of 2.53 nM (Fig. 3k), which was similar to the binding affinity of Clz with hCB1R (Supplementary Fig. 5e, *K*d = 2.65 nM). The binding affinities were comparable with that of AEA to hCB1R (Supplementary Fig. 5f, *K*d = 4.10 nM). These studies illustrated the competitive binding mode between AP drugs and the endocannabinoids when binding to CB1R.

CB1R is an G protein-coupled receptor that undergoes agonist-induced internalization after prolonged agonist exposure.^20^ We then detected the trafficking of CB1R upon AP drug treatments. It was found that CB1R was confluent in cytoplasm in high doses of Olz treatments (Fig. 3l, m) and in Clz-treated myocytes (Fig. 3m), while intact cells showed prominent membrane-location of CB1R (Fig. 3m, white arrowheads). Three sites have been reported to associate with CB1R protein trafficking^21,22^ and were thus mutated (C416A, S426A, and S430A), respectively. To clarify which site is responsible for AP drugs-induced CB1R internalization, we established a *Cb1r* knockout (*Cb1r*^*−/−*^) cell line using CRISPR-guided gene editing. The *Cb1r*^*−/−*^ cells were then transfected with each of these mutation plasmids. It was found that the mutant C416A did not affect the Olz or Clz-induced CB1R internalization; However, when S426 or S430 was mutated, CB1R showed a large proportion of membrane-localization (Fig. 3n, arrowheads), indicating the necessity of S426 or S430 sites for CB1R internalization by AP drug treatments.

To further confirm the pyroptosis-based dual-omics screen results, we transiently transfected a recombined *Cb1r* overexpression (CB1R-OE) plasmid, or a short-hairpin RNA-delivered silencing plasmid of *Cb1r* (shCB1R) into mouse HL-1 cells (Supplementary Fig. 5g). However, neither the CB1R-OE plasmid nor shCB1R plasmid regulated the mRNA levels of pyroptosis genes *Nlrp3*, *Asc*, *Casp1*, and *Gsdmd* (Supplementary Fig. 5h–k). We then established HL-1 cells stably expressing *Cb1r* using a lentivirus system (Lv-CB1R). It was detected that overexpression of *Cb1r* enhanced the protein levels of NRLP3, ASC, Casp1 and GSDMD, while specific CB1R antagonists (AM 251, AM 281, and Rimonabant) dampened Olz-induced expression of pyroptosis markers (Fig. 3o). Likewise, while a selective CB1R agonist ACEA alone or in combination with AP drugs (Olz and Clz) significantly promoted pyroptosis protein expression in *Cb1r*^+/+^ cells (hereafter termed as WT cells), knockout of *Cb1r* (*Cb1r*^*−/−*^) decreased the above proteins to a barely detectable level (Fig. 3p). In the time-lapse microscopy, pretreatments with the CB1R antagonist AM 281 significantly abolished Olz or Clz-induced cell pyroptotic protrusions (arrows) and PI uptake (Fig. 3q, r). All these results strongly suggested that CB1R is an upstream regulator of the NLRP3 inflammasome.

### CB1R interacted with NLRP3 inflammasome via amino acid residues 177–209 and prevented it from degradation

**Fig. 4:**
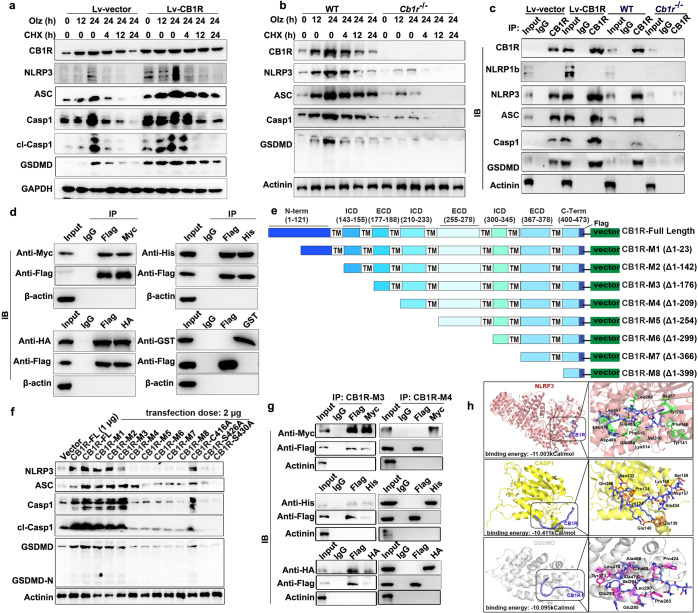
CB1R assembled multiple pyroptosis proteins via amino acids 177–209 and prevented the NLRP3 inflammasome from degradation. **a**, **b** In the *Cb1r* knockout HL-1 cells or the *Cb1r* stably overexpressed HL-1 cells, Olanzapine (Olz, 4 μM) with or without the specific protein synthesis inhibitor (cyclohexane, CHX, 200 nM) were added to treat cells for indicated hours. Western blot was performed to detect the protein levels of pyroptotic proteins. **c** In the *Cb1r* knockout HL-1 cells or the *Cb1r* stably overexpressed HL-1 cells, co-immunoprecipitation (Co-IP) assay was performed to detect the endogenous physical interaction between CB1R with pyroptotic proteins. **d** A flag-tagged *Cb1r* plasmid was co-transfected with each of the Myc-tagged *Nlrp3* plasmid, His-tagged *Gsdmd* plasmid, HA-tagged *Casp1* plasmid, and the GST-tagged *Asc* plasmid in HL-1 cells. Co-IP assay was performed to detect exogenous interaction of CB1R with the NLRP3 inflammasome. **e** Schematic illustration of mouse CB1R with full length (CB1R-FL, 1-473 amino acids), and truncated domains including Δ1-23 (CB1R-Mutant 1, or CB1R-M1), Δ1-142 (CB1R-M2), Δ1-176 (CB1R-M3), Δ1-209 (CB1R-M4), Δ1-209 (CB1R-M5), Δ1-254 (CB1R-M5), Δ1-299 (CB1R-M6), Δ1-366 (CB1R-M7), Δ1-399 (CB1R-M8). **f** The CB1R-FL, eight truncation mutants and three point-mutants were individually transfected into HL-1 cells. Western blot assay was performed to detect the protein levels of pyroptotic proteins. **g** The CB1R-M3 and CB1R-M4 plasmids were individually transfected into HEK293T cells in combination with each of the recombinant pyroptosis plasmids. Co-IP assay was conducted to detect protein interaction. **h** The simulated interaction diagram of human CB1R peptide (amino acids 177-189) with human proteins NLRP3 (up, pink cartoon), CASP1 (middle, yellow cartoon), or GSDMD (down, gray cartoon) in left panels. Right panels showed the CB1R peptide (blue) and key NLPR3 (green), CASP1 (orange), and GSDMD (purple) residues involved in ligand binding in stick representation

Given CB1R dominated the pyroptosis molecule at protein levels, we then used cyclohexane (CHX, 200 nM), an inhibitor of protein synthesis, to study whether CB1R affected pyroptosis protein synthesis or degradation. It was found that major NLRP3 inflammasome components gradually degraded in control cells after 4 h’ CHX co-treatment with Olz. When CB1R was stably overexpressed, these pyroptosis proteins were still detectable after 12 or even 24 h’ CHX co-treatments (Fig. 4a). In the *Cb1r* knockout myocytes, Olz marginally increased the expression of NLRP3, ASC, Casp1, and GSDMD, and co-treatment with CHX decreased more evidently these pyroptosis proteins (Fig. 4b). These observations suggested that CB1R stabilized the NLRP3 inflammasome by inhibiting their degradation.

In view of CB1R internalization after AP drug treatments, we then performed immunoprecipitation assay to assess whether CB1R binds directly with the NLRP3 inflammasome. In the control cells, precipitates by a CB1R antibody were detected of NLRP3, ASC, Casp1, and GSDMD, but not NLRP1b, reinforcing the interaction between CB1R and NLRP3 inflammasome. The CB1R antibody-precipitated pyroptosis proteins were further enhanced in the Lv-CB1R cells when comparing with the Lv-vector cells, and were remarkably suppressed in the *Cb1r*^*−/−*^ cells when comparing with the WT cells (Fig. 4c). Further, co-immunoprecipitation assays showed that the precipitate from anti-Flag-CB1R antibody was detected of the Myc-NLRP3 protein, His-GSDMD protein, and HA-Casp1 protein individually, but not the GST-tagged ASC, and vice versa (Fig. 4d). In the mouse hearts, immunofluorescence assays also showed that CB1R co-localized with NLRP3 in the Olz or Clz-treated mice (Supplementary Fig. 5l). These data suggested the direct physical interaction of CB1R with NLRP3, Casp1, and GSDMD with exception for ASC.

To further decipher which domain of CB1R was responsible for the regulation of the NLRP3 inflammasome, we constructed serial truncations of the Flag-tagged CB1R plasmid (Fig. 4e). It was found that the CB1R-FL plasmid promoted the pyroptosis proteins in a transfection dose-dependent manner. Transient transfection of the CB1R-M1, CB1R-M2, or CB1R-M3 mutants generally resembled the CB1R-FL plasmid effects (Fig. 4f). However, CB1R-M4 and the following truncation plasmids failed to maintain the pyroptosis protein levels (Fig. 4f). Moreover, transfection of the CB1R-C416A mutant, which retained the ability to internalize (Fig. 3n), also maintained the high abundance of pyroptosis proteins; whereas transfection of the CB1R-S426A or CB1R-S430A plasmid, two mutants that lose internalization capacity (Fig. 3n), failed to regulate the expression of the NLRP3 inflammasome (Fig. 4f), reinforcing the requirement of CB1R internalization for regulating the NLRP3 inflammasome expression. Co-immunoprecipitation assays also showed that the CB1R-M3 mutant, but not CB1R-M4, precipitated each of the Myc-NLRP3, His-GSDMD, and HA-Casp1 proteins (Fig. 4g), suggesting the functional importance of CB1R sequences 177–209. Within the CB1R sequences 177–209, the region 177–189 locates at the extracellular domain (ECD) and may have potential protein interactive activity. Indeed, molecular docking analysis confirmed interactions of the CB1R region 177–189 with each of the human NLRP3, CASP1, and GSDMD proteins (Fig. 4h). All these data suggested that CB1R stabilized the NLRP3 inflammasome *via* direct interaction with the major inflammasome components after internalization.

### Genetic knockout of *Cb1r* ameliorated AP drugs-induced pyroptosis and cardiotoxicity

**Fig. 5:**
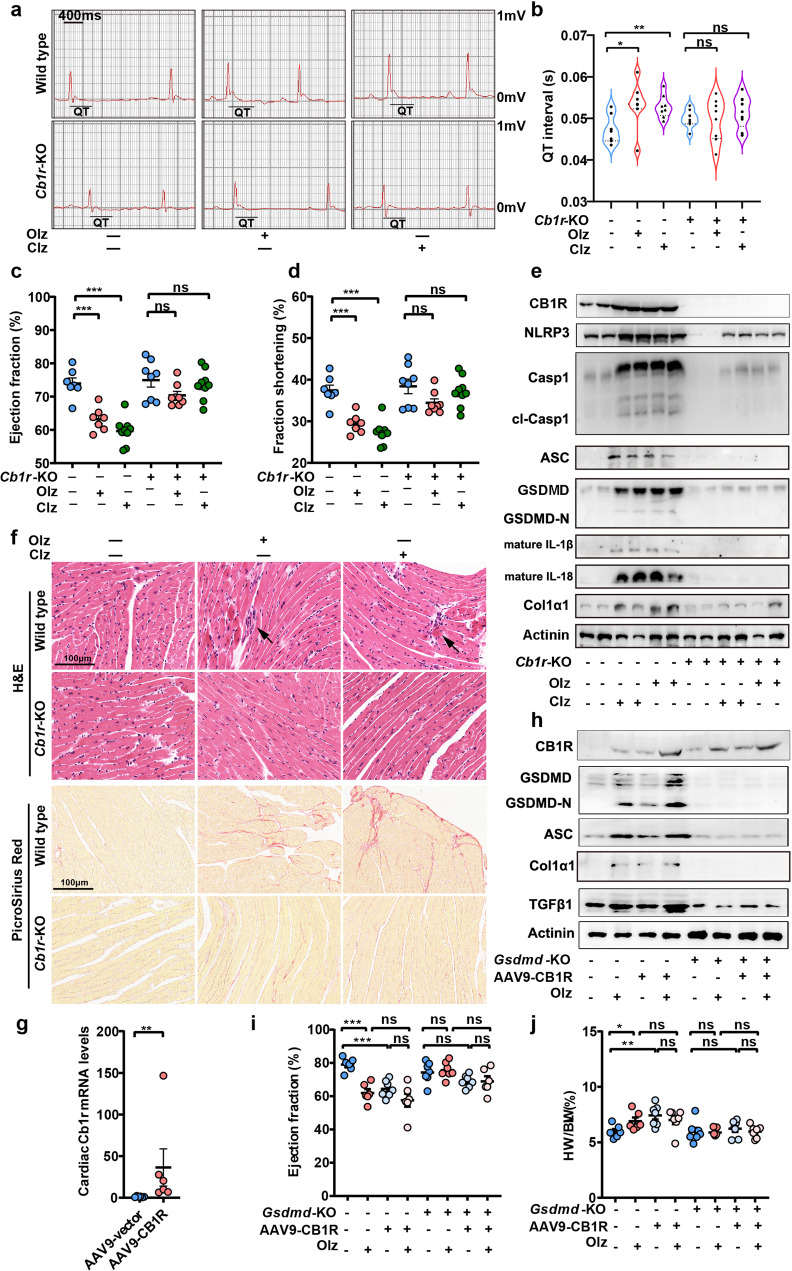
Genetic knockout of Cb1r alleviated antipsychotics-induced pyroptosis and cardiotoxicity. **a**, **b** Electrocardiography (ECG) monitoring of mice (n = 7–9 mice/group). **c**, **d** Echocardiography assessment of left-ventricle function of mice (*n* = 7–9 mice/group). **e** Western blot analysis of CB1R, NLRP3 inflammsome, and pro-fibrotic factors expression. **f** Histological analyses of hearts from each group of mice. Arrows indicated inflammatory infiltrate and hypereosinophilia of cardiomyocytes. Scale bar = 100 μm. **g** qRT-PCR analysis of the efficacy of cardiac overexpression of *Cb1r* using the AAV9 system (*n* = 6/group). **h** Western blot analysis of pyroptosis proteins and pro-fibrotic factors in WT and *Gsdmd*^*−/−*^ mice receiving indicated treatments. **i**, **j** Ejection fraction was detected and heart weight (HW) was measured for the indicated treatments. All quantification represents the mean and SEM of each group. Unpaired Student’s *t*-test was used for analysis in (**g**). Two-way ANOVA was used for analysis in (**b**–**d**, **i**, **j**). ns, no significance. *p < 0.05; **p < 0.01; ***p < 0.001 as indicated

We then genetically depleted the *Cb1r* in mice. Electrocardiography revealed that the average QT interval was significantly prolonged by chronic Olz or Clz treatments in WT mice. However, when *Cb1r* was depleted from heart, both Olz and Clz no longer prolonged the QT interval (Fig. 5a, b). While WT mice showed heart dysfunction after long-term Olz and Clz stimuli, *Cb1r*^*−/−*^ mice showed normalized heart function even under drug stimuli, as manifested by left-ventricle EF and FS (Fig. 5c, d). Western blotting showed that both Olz and Clz promoted the cardiac CB1R expression, accompanying with enhancement of the NLRP3 inflammasome, mature pro-inflammatory factors, and pro-fibrotic factors in WT but not the *Cb1r*^*−/−*^ mouse hearts (Fig. 5e). Histologically, when Olz or Clz induced hypereosinophilia of myocardium and unwelcome cardiac remodeling such as inflammatory cell infiltrates (black arrows) and the accumulation of fibrotic tissues in WT mice, genetic knockout of *Cb1r* remarkably inhibited these pathological changes (Fig. 5f).

We also constructed an AAV9-delivered *Cb1r* overexpression plasmid and in situ injected this expression system into mouse hearts (Fig. 5g). We observed that *Cb1r* overexpression alone induces the cardiac expression of pyroptosis and pro-fibrotic factors (Fig. 5h) and significantly damped left-ventricle function while promoting HW gain in WT mice (Fig. 5i, j). However, in the *Gsdmd*^*−/−*^ mice, the AAV9-CB1R plasmid alone or in combination with Olz treatments no longer induced the above effects (Fig. 5h–j). These genetic studies suggested the GSDMD-dependent regulation of AP drug cardiotoxicity by CB1R.

### Pharmacologic inhibition of CB1R protected mice from AP drug cardiotoxicity without causing additional side effects

**Fig. 6:**
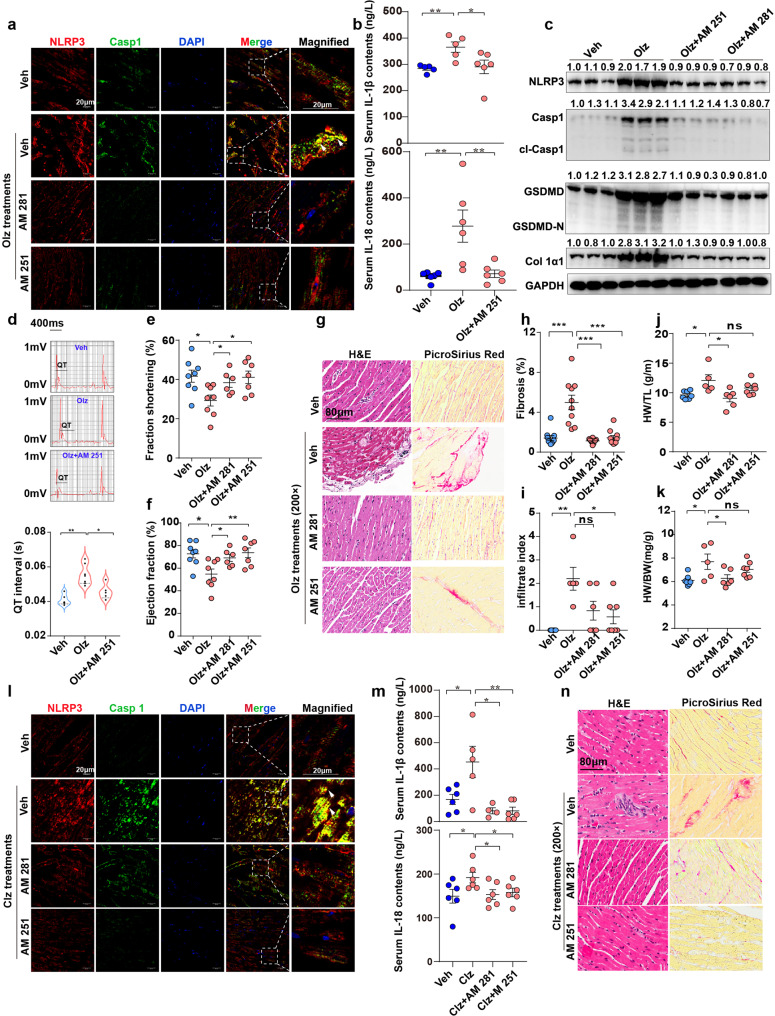
Pharmacologic inhibitors of CB1R rescued antipsychotics-induced pyroptosis and cardiotoxicity. **a** Immunofluorescence staining of NLRP3 and Casp1 in Olz (5 mg/kg)-treated mouse hearts. Arrowheads indicate NLRP3 specks (yellow signal). Scale bar = 20 μm. **b** ELISA detection of mouse serum IL-1β and IL-18 contents (*n* = 5–6/group). **c** Western blotting of pyroptotic protein levels. The values above each protein blot represent the ratio of protein intensity to tubulin. **d** ECG monitoring of QT interval (*n* = 5–7/group). **e**, **f** Echocardiography measurements of ejection fraction and fraction shortening (*n* = 5–8/group). **g** H&E staining and PicroSirius red staining of hearts tissues under indicated treatments. Scale bar = 80 μm. Quantitative analysis of fibrosis, infiltrate index, heart weight/body weight (HW/BW), and HW/right tibia length (HW/TL). *n* = 10 in (**h**) and *n* = 5–7 in (**i**–**k**). **l** Immunofluorescence staining of NLRP3 and Casp1 in Clz (25 mg/kg)-treated heart tissues. Arrowheads indicate NLRP3 specks (yellow signal). Scale bar = 20 μm. **m** ELISA detection of mouse serum IL-1β and IL-18 contents (*n* = 5–6/group). **n** H&E staining and PicroSirius red staining of hearts tissues under indicated treatments. Scale bar = 80 μm. All quantification represents the mean and SEM of each group. Unpaired Student’s *t*-test was used for analysis in (**b**, **d**, **e**, **f**, **h**–**k**, **m**). ns, no significance. *p < 0.05; **p < 0.01; ***p < 0.001 as indicated

Next, the ability of CB1R inhibitors to recapitulate the protection observed in the genetic model was explored. Antagonists of CB1R (AM 251 and AM 281) were tested for in vivo therapeutic efficacy. With AM 251 or AM 281 pretreatments, the fluorescence of NLRP3 or Casp1 was blurred, and the number of inflammasome specks was notably decreased (Fig. 6a). As a reflection of the impaired inflammasome activity, serum IL-1β and IL-18 levels were consistently decreased by the AM 251 co-treatments (Fig. 6b). The CB1R antagonists also conferred strong suppression of the cardiac pyroptotic proteins and fibrotic factor Col1α1 expression (Fig. 6c). As a consequence of pyroptosis suppression, Olz-prolonged QT interval was rescued by AM 251 pretreatments (Fig. 6d). AM 251 or AM 281 also significantly improved left-ventricle function (Fig. 6e, f), heart histopathology (Fig. 6g–i), and decreased HW (Fig. 6j, k). Likewise, AM 251 or AM 281 was also observed to inhibit the formation of NLRP3 inflammasome specks (Fig. 6l) and decreased the serum levels of IL-1β and IL-18 (Fig. 6m) in Clz-treated mouse hearts. No remarkable inflammation or fibrosis was observed in AM 251 or AM 281 co-treated mice, a result that was in great contrast to sole Clz-treated mouse hearts (Fig. 6n).

To assess whether the above CB1R antagonists induced additional side effects, we monitored serum glucose, TG, and TC levels. It was shown that Olz did not elevate serum metabolic parameters in 21 days, co-treatment with AM 251 or AM 281 did not affect these parameters either (Supplementary Fig. 6a–c). These three antagonists did not impose significant in vitro toxicity to myocytes within the 0.01–10 μM dose ranges (Supplementary Fig. 6d–f) or within 60 h’ exposure time (Supplementary Fig. 6g–i). Co-treatment of individual CB1R antagonist with Olz did not enhance electrocardiographic changes with exception for Rimonabant which induced remarkable elevation of the J-wave amplitude (Supplementary Fig. 6j). Co-treatments with AM 251 or AM 281 did not cause concrete damages to solid organs (Supplementary Fig. 6k–p). These observations suggested that AM 251 and AM 281 might serve as safer agents while defending the heart from antipsychotics cardiotoxicity.

### Expression of CB1R positively correlated with extents of AP drugs-induced pyroptosis and fibrosis in human heart specimens

**Fig. 7:**
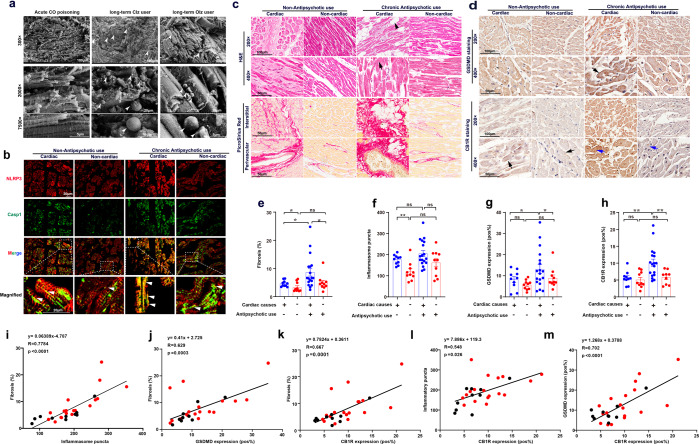
CB1R expression positively correlated with antipsychotics-induced pyroptosis and fibrosis in human specimens. **a** Scanning electron microscopy of hearts from a control (acute CO poisoning) case and two antipsychotic drug (Clz, Olz) users. Arrowheads indicates bubble-like pyroptotic protrusions. **b** Immunofluorescence analysis of NLRP3 (red) and Casp1 (green) in the four categories of human heart specimens. Arrowheads indicated NLRP3 inflammasome specks (yellow signal). Scale bar = 20 μm. **c** H&E staining and PicroSirius red staining of human heart sections. Black arrowhead indicated fatty-fibro tissues and black arrow indicated contraction band necrosis. Magnification = 200× for upper images and 400× for lower images at each staining. **d** Immunohistochemistry staining of GSDMD and CB1R within the human heart sections. Black arrows indicated membrane-gathering, whereas blue arrows indicated cytoplasm-localization. **e**–**h** Quantitative analysis of fibrosis, inflammasome specks, GSDMD expression, and CB1R expression within the heart sections under 400× (*n* = 18 for antipsychotics users with cardiac deaths, and *n* = 10 for the other three groups). **i**–**m** Linear correlation analysis. Red dots indicated antipsychotics users with cardiac deaths (*n* = 18) and black dots indicated antipsychotics users with non-cardiac deaths (*n* = 10). All quantification represents the mean and SEM of each group. Two-way ANOVA with Bonferroni post-hoc test was used for analysis in (**e**–**h**). ns, no significance. *p < 0.05; **p < 0.01 as indicated

The clinical translatability of CB1R-regulated NLRP3 inflammasome was evaluated next. In authentic autopsy cases, scanning electron microscopy showed that the heart from an acute carbon monoxide (CO) poisoning case displayed well arrangement of myofiber without remarkable protrusions around cell surface; however, in a long-term Clz or Olz user, multiple protrusions in distinct sizes were observed to attach the myofibers (Fig. 7a). Heart specimens from four categories of decedents who were age and gender matched were then collected (Supplementary Table 1). Immunofluorescence assay showed that antipsychotic users with cardiac deaths presented with remarkable NLRP3 inflammasome specks (Fig. 7b, white arrowhead). Cardiomyocytes in this group were surrounded by a halo of fibro-fatty tissues (black arrowhead) and showed sporadic contraction band necrosis (black arrow) and substantial fibrotic tissue accumulation (Fig. 7c). Furthermore, we observed intense staining of GSDMD and CB1R in antipsychotic users with cardiac deaths, with GSDMD strongly localized to membrane and CB1R mostly localized to cytoplasm (Fig. 7d). Antipsychotic users with cardiac deaths presented the most severe cardiac fibrosis, highest number of NLRP3 inflammasome specks, and highest expression of both GSDMD and CB1R as compared with the other three groups (Fig. 7e–h). Linear regression analysis of the 28 antipsychotic users revealed that the number of NLRP3 inflammasome specks (Fig. 7i) or GSDMD expression (Fig. 7j) significantly correlated with extents of heart fibrosis. CB1R expression showed good correlation with the extent of fibrosis (Fig. 7k), number of the NLRP3 inflammasome specks (Fig. 7l), and GSDMD expression (Fig. 7m). Overall, these data provided profound evidence that aberrant expression of CB1R correlated with antipsychotics-induced pyroptosis and fibrosis in human hearts.

## Discussion

The present study uncovered the NLRP3 inflammasome-mediated pyroptosis as a major player of AP drugs-evoked cardiotoxicity and identified CB1R as a critical regulator of the NLRP3 inflammasome stability. We proposed CB1R or NLRP3 inflammasome inhibition as effective means of rescuing the cardiac side effects of AP drugs.

Despite major recognition of AP drugs-elicited metabolic and cardiovascular adverse effects,^2,3^ few studies have compared the time effects of AP drugs-induced metabolic and cardiac outcomes. Using a clinical comparable dose of the antipsychotic Olz, we have provided concrete evidence that cardiotoxicity is induced preceding the development of systemic metabolic disorders. Within the 21 days’ consecutive treatments, QT interval prolongation, a condition predisposing to sudden cardiac death,^13^ was observed early after 14 days. Both Olz and Clz tremendously accumulated in mouse heart at 21 days, a time point when metabolic organs and other solid organs did not develop remarkable aberrance. Lysosomes are responsible for metabolism of drugs and heart is featured as a lysosome-deficient organ. Therefore, the heart-restricted toxicity conformed to the notion that AP drugs accumulate in lysosome-poor organs such as heart but could be eliminated in lysosome-rich organs such as liver and kidney.^23^ In line with our data, a previous study observed that Olz-induced hyperinsulinemia and hypertriglyceridemia were evident until 8 weeks of treatment.^24^ These data could explain why some patients with psychosis suffer from premature cardiac deaths before the development of obesity and diabetes, with an average of 25 years shortened life than general population.^25^ The precedent development of cardiotoxicity thus highlighted early monitoring after AP drugs administration. Unfortunately, proposed early diagnostic methods, such as circulating microRNAs,^26^ N-Terminal fragment of B-Type Natriuretic Peptide (NT-pro-BNP) alone^27^ or in adjunction with QTc measurement^28^, and routine electrocardiogram monitoring as a prerequisite^29^ have proven to be ineffective^26^ or been debated.^30^ Therefore, alternative intervention or therapeutic strategies are mandated for AP drugs-induced cardiotoxicity.

Using a transcriptomic analysis, we have identified that the NLRP3 inflammasome-mediated pyroptosis underlined AP drugs cardiotoxicity. Other types of programmed cell death were not as critical as pyroptosis that drove the drug cardiotoxicity. These findings were reinforced when pretreatments of a specific NLRP3 inhibitor MCC950^31^ or a specific Casp1 inhibitor VX-765 (Belnacasan),^32^ genetic knockout of *Nlrp3* or *Gsdmd* significantly dampened Olz-induced cardiotoxicity. AP drug use has been well linked with inflammatory activity,^33^ with reports of increased number of deaths from autoinflammation diseases such as myocarditis and pneumonia after long-term APs use.^14^ Our data showed that Olz triggered both the priming and activation signaling of pyroptosis in a time- and dose-dependent manner. Genetic ablation or pharmacologic inhibition of pyroptosis successfully eliminated the detrimental effects caused by AP treatments, opening a promising avenue for cell death-based treatment of diseases, especially because VX-765, the Casp1 inhibitor used in this study,^32^ is currently undergoing a phase II clinical evaluation.

As AP drugs are lipophilic, they are assumed to perturb biological membrane and bind with membrane receptors^23^ before transducing extracellular stress into cellular signaling (pyroptosis). However, we have previously shown that it is hard to capture the core membrane receptors by using single proteome-based screen due to technical limitations.^34^ To fill the gap between AP drug stimulation and pyroptosis activation, we thus performed a pyroptosis-based dual-omics screen and identified CB1R, a GPCR physiologically locating at the biological membrane, as a critical stabilizer of the NLRP3 inflammasome. CB1R is a major regulatory machinery from the endocannabinoid system, which handles lipid signal transduction.^35^ We have recently reported a clue that cannabinoid receptors might be involved in Clz^17^ or Que cardiotoxicity^18^ with CB1R and CB2R conferring opposite effects.^36^ The interactive mode of AP drugs with CB1R and the intracellular signals following CB1R activation have remained yet to be elucidated. Herein, we identified that AP drugs are previously unappreciated ligands that competitively bind to CB1R at strikingly nanomolar levels of affinity. Upon direct stimulation by AP drugs, CB1R regulated the NLRP3 inflammasome activity by stepwise processes, namely an initial translocation from membrane to cytoplasm *via* phosphorylation at the S426 or S430 sites and a followed physical interaction with the NLRP3 inflammasome components *via* its amino acids 177–209. In human cases, cardiac CB1R expression correlated with extents of pyroptosis and fibrosis elicited by AP drug uses, a profound implication for the clinical translatability of CB1R inhibition. In general, antagonists of CB1R have conferred promising clinical prospects, particularly in cardiovascular protection,^37,38^ whereas agonists of CB1R (e.g., marijuana and synthetic cannabinoids) have been shown to cause serious adverse cardiovascular events.^39^ In this study, we found that selective CB1R antagonists AM 251 and AM 281 were protective against AP drug cardiotoxicity without causing additional toxicity to metabolic or solid organs. However, the CB1R antagonist Rimonabant was an exception that caused additional arrhythmia in mice. This was basically in line with the fact that the use of Rimonabant causes additional toxicity such as serious psychiatric disorders,^40^ making this weight-loss drug discarded from the market. In general, CB1R antagonists have evolved from the first-generation brain penetrance (e.g., Rimonabant) to second-generation peripherally restricted neutral antagonists, and to a third-generation multi-targeting (poly-pharmacology) strategy.^41^ Though AM 251 and Rimonabant share structural similarity and are both brain-penetrating CB1R antagonists, AM 251 possesses other potential targets such as GPR55^42^ which might decrease the risk of further toxicity^43^ and thus explains the differences in their cardiac actions. Moreover, the peripherally restricted CB1R antagonists devoid of intracranial side effects, such as AM6545^44^ or JD-5037^45^ have been shown to largely improve peripheral metabolic damages. In view of both metabolic and cardiac side effects associated with long-term antipsychotics use, these CB1R antagonists, together with some first-generation CB1R antagonists (i.e., AM 251 and AM 281), might thus confer dual actions in clinical practice: one to protect against AP drug-induced cardiotoxicity (as evidenced by the present study), and the other to improve AP drug-induced metabolic disorders in longer-term medication. Therefore, the selection of low-toxic CB1R antagonists without compromising therapeutic effects, particular those with cardiac and metabolic dual actions, is critical for developing cardioprotectants for AP drug users. Testing the safety and efficacy of these low-toxic antagonists would merit further investigation in preclinical studies.

The identification of CB1R-activated NLRP3 inflammasome-mediated pyroptosis as a major driver of AP drugs-evoked cardiotoxicity is of paramount importance. First, currently, the rescue strategy has been only drug-withdrawal or discontinuation when situated for AP drugs-induced dysrhythmia. This passive action, unfortunately, leads to the economic waste of on-the-market drugs. Alternatively, the development of CB1R antagonists with both efficacy and safety in clinical trials would largely promote the marketed use of efficacy-potent AP drugs without worrying the unwelcome cardiotoxic effects and thereby saving the economic loss by drug withdrawal. Second, multiple upstream signals, including deubiquitination, efflux of potassium ions (K^+^) or chloride ions (Cl^−^), flux of calcium ions (Ca^2+^), lysosomal disruption, mitochondrial dysfunction, metabolic changes, and trans-Golgi disassembly, are thought to regulate the NLRP3 activation.^46^ We found that after binding to AP drugs, CB1R internalized to cytoplasm where its 177–189 amino acid domain had spatial proximity to and directly interacted with the pyroptosis proteins, a mechanism that prevented the inflammasome from degradation. We hypothesized that internalized CB1R might recruit co-regulators such as deubiquitin ligases to aid in suppressing the NLRP3 inflammasome degradation. Meanwhile, the CB1R 177–189 region-dependent regulatory mechanism explains why intact myocytes (the CB1R 177–189 domain localizes extracellularly and is thus separated from the intracellular inflammasome) are less susceptible to pyroptosis in physiological condition, while chronic use of cannabinoids and cannabis extracts commonly elicits inflammasome-related human diseases.^47^ The evidence of CB1R trafficking to cytoplasm and physically interacting with the NLRP3 inflammasome components, as reported here, also reinforced the involvement of GPCRs in the activation of NLRP3 inflammasome and opened new avenue of GPCRs as drug targets for the therapy of NLRP3-driven diseases.^48^ Third, though CB1R is now considered to be ubiquitously expressed across the bodies^49^ and Olz might bind to CB1R in other solid organs harboring CB1R expression, Olz exclusively concentrated in the heart and caused toxic effects. Therefore, intense monitoring of the heart concentration, instead of blood concentration of AP drugs, might better guarantee drug safety.

**Fig. 8:**
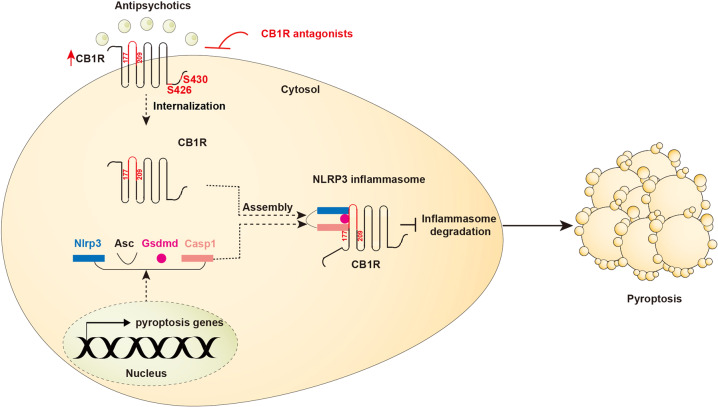
Schematic illustration of CB1R-stabilized NLRP3 inflammasome to drive antipsychotics-induced cardiomyocyte pyroptosis. Upon chronic antipsychotics stimuli, CB1R internalized *via* S426 and S430 sites and physically interacted with the NLRP3 inflammasome through its region 177–209, and thereby preventing degradation of the NLRP3 inflammasome that consequently activated pyroptotic cell death and eventually contributed to antipsychotic drugs cardiotoxicity. CB1R inhibitors with low additional toxicity represent attractive approaches when treating the NLRP3-driven diseases

Overall, we have provided concrete evidence that the NLRP3 inflammasome-mediated pyroptosis drove AP drugs cardiotoxicity. The NLRP3 inflammasome activity was controlled by CB1R, which internalized *via* S426 and S430 and physically interacted with the NLRP3 inflammasome through its region 177-209, leading to stabilization of the NLRP3 inflammasome and activation of pyroptotic cell death that eventually contributed to AP drugs cardiotoxicity (Fig. 8). CB1R inhibitors with low additional toxicity (i.e., AM 251, AM 281, and peripherally-restricted antagonists) represent attractive approaches when treating the NLRP3-driven diseases.

## Materials and methods

### Human subjects

The decedents’ next-of-kin provided informed consent granting the use of heart samples and associated data and images in this publication according to the requirements set forth by the Institutional Review Board at the School of Basic Medical Sciences, Fudan University (grant No.: 2020-009). Please refer to Supplementary Table 1 for information about the decedents’ age, sex, and disease details. Histologic analyses of human specimens were performed in a blinded manner by three independent pathologists.

### Mouse models

Key resources including antibodies, experimental organisms, software, commercial kits, and primer sequences were listed in Supplementary Table 2 for detail. Wild type (WT) C57BL/6 or Balb/C mice were purchased from Shanghai Laboratory Animal Center (Shanghai, China). The *Gsdmd* knockout (*Gsdmd*^*−/−*^ or *Gsdmd*-KO) mouse was a generous gift from Professor Feng Shao from the National Institute of Biological Sciences, Beijing, China. *Cb1r* knockout (*Cb1r*^*−/−*^) mice on the background of C57BL/6 were generated by the Shanghai Model Organisms (Shanghai, China). *Nlrp3* knockout (*Nlrp3*^*−/−*^ or *Nlrp3*-KO) mice on the background of C57BL/6 were in house breeding. Animal studies were conducted in accordance with the guidelines from the Institutional Animal Care and Use Committee at the School of Basic Medical Sciences, Fudan University (No.: 20170223-004). All efforts were made to minimize animal suffering. All histologic analyses were performed in a blinded manner.

### RNA sequencing and proteomic analysis

Total RNAs were isolated from control and Olz-treated hearts (*n* = 3/group) using Trizol solution (Vazyme, Nanjing, China) following the manufacturer’s instructions. To construct the RNA-seq library, 2 μg of total RNA per sample was mixed with poly-T oligo attached magnetic beads to isolate poly-A mRNA following mRNA fragmentation. The cleaved RNA fragments were transcribed into cDNAs, which were then purified using AMPure XP beads to remove all reaction components. Transcript quantification of RNA-seq reads were performed with Genomic Alignments (ver.1.20.1) by reads aligned to Ensemble Mus Musculus transcriptome annotation (GRCm.38. p5). The FPKM values were calculated using ‘fpkm’ function from DESeq2 (ver. 1.24.0) that were processed with the robust median ratio method and transcript reads were normalized by the ‘voom’ function from Limma (ver. 3.40.6). To assess whether a transcript was differentially expressed, EdgeR (ver. 3.24.3) calculates the results based on the normalized counts from entire sequence alignments. Differentially expressed (DE) genes were defined as a fold change of raw FPKM value > 2 and adjusted *p* value < 0.01. Control and Olz-treated hearts were also processed for label-free proteomic analysis. Proteomics sample preparation and mass spectrometry analysis was performed in MaxQuant as described previously.^50^ Label-free quantification (LFQ) of proteins were compared between control and Olz-treatment groups using Student’s t test, and DE proteins were clustered when the yielded *p* value was <0.05 and fold change was >1.5.

### Molecular docking

The ligand-CB1R binding mode was analyzed by molecular docking. We downloaded the crystal structure model of CB1R (PDB ID:5TGZ) from RCSB Protein Data Bank operated by the Brookhaven National Laboratory (https://www.rcsb.org). The 3D structure models of active ligands (Olz, Clz, AEA, and 2-AG) were built by Schrodinger 2015. All the dockings in this research were performed with Schrödinger’s Glide. We used the standard precision mode of Glide, which is efficient and accurate for most of the targets. Glide generates the possible binding modes of ligand–protein complexes and scores them with GlideScore, a mixture of interaction energy and parameter-based penalty functions that roughly represents binding energy.

For analysis of CB1R-pyroptosis proteins interaction, we extracted the peptide fragment of 179–188 amino acids of human CB1R and explored the interaction between the peptide and human CASP1 (PDB ID: 6PZP), NLRP3 (PDB ID: 6NPY), and GSDMD (PDB ID: 5NH1) proteins. We downloaded the crystal structure models of these proteins from https://www.rcsb.org and used glide module in Schrodinger 2015^51,52^ to investigate the binding models of the CB1R peptide fragment in these three proteins.

### Statistical analysis

For correlation analysis, linear regression models were performed and the goodness of fit for regression models was assessed using R values. For numerical variables, a two-tailed Student’s *t*-test was used for comparison of means between groups, while analysis of variance (ANOVA) was used for comparisons of ≥3 groups, followed by a Bonferroni post hoc test. Normal distribution was initially analyzed and then Brown–Forsythe test was used for the analysis of homogeneity of variance when necessary. The Student’s *t*-test was used to compare the means of the initial data after normal distribution, and Wilcoxon signed rank test was used to study data after non-normal distribution. All group numbers and detailed significant values were presented within the figure or their legends. A *p* value less than 0.05 was considered as statistical significance. GraphPad Prism 8.0 (La Giolla, CA, USA) was used for statistical analysis and graphing.

## Supplementary information

Supplementary materials

Supplementary video 1

Supplementary video 2

Raw western blots

## Data Availability

The mass spectrometry proteomics and RNA-seq data have been deposited to the ProteomeXchange Consortium (http://proteomecentral.proteomexchange.org) *via* the iProX partner repository with the dataset identifier PXD030588.
