# Supplementary material for: CB1R-stabilized NLRP3 inflammasome drives antipsychotics cardiotoxicity
Source: Signal Transduct Target Ther. 2022 Jun 24;7:190. doi: 10.1038/s41392-022-01018-7 (PMC9225989; doi:10.1038/s41392-022-01018-7)
Supplement: Supplementary file 1 — Supplementary materials [file 41392_2022_1018_MOESM1_ESM.docx]

**Supplementary Materials for**

CB1R-stabilized NLRP3 inflammasome drives antipsychotics cardiotoxicity

Liliang Li^1,2, #, *^, Pan Gao^3, #^, Xinru Tang^1^, Zheng Liu^1^, Mengying Cao^3^, Ruoyu Luo^2^, Xiaoqing Li^1^, Jing Wang^1^, Xinyi Lin^1^, Chao Peng^4^, Zhihong Li^4^, Jianhua Zhang^5^, Xian Zhang^6^, Zhonglian Cao^7^, Yunzeng Zou^3, *^, Li Jin^2, 8, *^

Correspondence to: [liliangli11@fudan.edu.cn](mailto:liliangli11@fudan.edu.cn); [zou.yunzeng@zs-hospital.sh.cn](mailto:zou.yunzeng@zs-hospital.sh.cn); [lijin@fudan.edu.cn](mailto:lijin@fudan.edu.cn).

These authors contributed equally: Liliang Li, Pan Gao.

**This PDF file includes**:

Supplementary materials and methods,

Supplementary Table 1 to 2,

Supplementary Figure 1 to 6

Supplementary Video 1 to 2

**Supplementary materials and methods**

**Mouse treatments**

To mimic the clinical scenario, the maintenance doses of olanzapine (Olz) and clozapine (Clz) used in the clinic were converted to appropriate doses in mice. Briefly, male Balb/C or C57BL/6 mice at the age of ~4 weeks were acclimated for 1 week. At 15:00 of each day, mice were intraperitoneally (I.P.) injected with 100 μL of vehicle (PBS, Veh group), Olz (5 mg/kg) or Clz (25 mg/kg). Protocols for establishing antipsychotics cardiotoxicity models were as previously described.^1,2^

To inhibit NLRP3 inflammasome-mediated pyroptosis, Balb/C mice were co-treated with antipsychotics and MCC950 (a specific NLRP3 inhibitor) or VX-765 (a Casp1 inhibitor) for 21 days. MCC950 (20 mg/kg) or VX-765 (50 mg/kg) was pretreated 1 h prior to antipsychotics I.P. injection on each day. Mice with pyroptosis gene knockout were respectively bred till 6 weeks old, followed by consecutive I.P. injection with PBS, Olz, or Clz for 3 weeks.

To study the effects of CB1R antagonists/agonists on Olz- or Clz-induced cardiotoxicity, Balb/C mice were pretreated with CB1R antagonists Rimonabant (4 mg/kg), AM 251 (2.5 mg/kg), or AM 281 (2.5 mg/kg) 1 h prior to antipsychotics *I.P.* injection.

To study the effects of *Cb1r* genetic ablation or overexpression on antipsychotic cardiotoxicity, *Cb1r^-/-^* mice or AAV9-mediated cardiac *Cb1r* expression mice were utilized. Briefly, WT mice and the *Cb1r^-/-^* mice were fed under the identical environment and received *I.P.* injection of antipsychotics for indicated periods. The AAV9 was used to construct the vector that drives expression of full length CB1R (amino acids 1-473, termed as AAV9-CB1R) (Hanbio Inc, Shanghai, China). AAV9-EGFP (enhanced green fluorescent protein) was used as a control. All adeno-associated viruses at 2×10^11^ vector genomes (vg) per mouse were *in situ* injected into 3-5 independent sites at left ventricles through a 31G catheter while mice were anesthetized using 2% isoflurane. Three weeks after AAV9 injection, mice were *I.P.* injected with antipsychotics for further 3 weeks as abovementioned.

For all animal experiments, mice were housed with ad libitum access to food and water in light- and temperature-controlled environments (12-hour/12-hour light/dark, 22°C). Mice weights were recorded on a daily basis before each injection unless otherwise stated. On the last day of treatment, all mice had blood collected via orbital vein to prepare serum samples, while at the same time, the right tibia length (TL) and heart weight (HW) were measured. Hearts were transversely dissected and part of the heart tissues was subject to formalin fixation and paraffin embedding for heart slicing. Liver samples were also embedded in Optimum Cutting Temperature (OCT) for oil red staining. The remaining heart tissues and other organs such as liver, left, lung, right kidney, whole brain and fatty tissues were stored at -80℃ until use.

**Cell lines and primary cultures**

Primary mouse cardiomyocytes were isolated according to protocols described previously.^3^ Primary cardiomyocytes, human AC-16, rat H9c2 and mouse HL-1 cells were cultured in DMEM supplemented with 10% FBS (Gibco, Carlsbad, CA, USA). Transient transfection of plasmids was conducted using lipofectamine 3000 (Invitrogen). The pLKO.1 and pLVX/puromycin lentiviral plasmids were used to construct stable expression system. For CRISPR-generated *Cb1r* knockouts, plasmids containing *Cb1r* guide sequences (*Cb1r^-/-^* cells) or empty backbone (WT cells) were transfected into mouse HL-1 cells. The following day, cells were detached by trypsinization, and GFP-positive cells were sorted individually into the wells of 96-well plates by FACS. *Cb1r* gene product was electrophoresed by PCR, followed by sequencing, while the CB1R protein depletion was validated by Western blot analysis. Finally, the validated *Cb1r*-knockout clonal lines were subsequently expanded. Mutation plasmids and PCR products were sequenced by Tsingke Biological Technology (Beijing, China).

**Electrocardiograph monitoring and echocardiograph measurements**

Electrocardiograph was monitored in an 8-min length by lead II assessment while mice were deeply anesthetized. The electrocardiograph (lead II) of mice was recorded using a multi-channel physiological signal system (RM-6240BD, Chengdu Instrument Factory, China) at a speed of 20 ms/div. QT interval (s) was then calculated. Since all mice were monitored at a heart rate of approximately 400bpm, we used QT interval without correction in the electrocardiographic recording. To assess the left ventricle function, echocardiography was performed while mice were anaesthetized with 2% isoflurane. The left ventricle was detected under the long‐axis M‐mode when heart rate was ~400bpm. Measurements of left ventricular end‐diastolic diameter (LVEDd) and left ventricular end-systolic diameter (LVESd) were recorded and transferred online to a computer for analysis. Ejection fraction (EF) and fraction shortening (FS) were calculated automatically using a Vevo 2100 High Resolution Imaging System (Visual Sonics Inc, ON, Canada).

**Histological analysis, morphometric analysis, and serum and urine chemistry**

Immediately after mouse sacrifice, organs were dissected and rinsed three times in saline. The organs were then dried on a paper tower and weighed before storage into a freezer. Mouse organs were then prepared for slides that were stained with hematoxylin & eosin (H&E) staining and PicroSirius Red staining to examine inflammatory infiltrates and fibrosis, respectively.

Serum chemistry, such as alanine aminotransferase (ALT), aspartate aminotransferase (AST), albumin, total triglyceride (TG), total cholesterol (TC) and glucose, and urine chemistry, such as blood urine nitrogen (BUN) and urine creatinine (Cr) were determined by Jiancheng Bioengineering Institute (Nanjing, China) by using a microplate reader (Biotek, Winooski, VT, USA).

**RNA isolation and quantitative PCR**

Total RNAs from cells or tissues were isolated using TRIzol solution (Invitrogen, Carlsbad, CA, USA). After assessment of RNA quality and concentration, an equal amount of RNA (500 ng) was reversely transcribed into cDNA using the HiScript II Q RT SuperMix (Vazyme, Nanjing, China). Quantitative real‐time PCR was performed in a LightCycler 480 Real‐Time System (Roche Diagnostics, Basel, Switzerland) using the AceQ qPCR SYBR Green Master Mix (Vazyme, Nanjing, China). *Gapdh*, *β-actin* and 18S rRNA were used as internal control as indicated. Primers were synthesized by Tsingke Biological Technology (Beijing, China) or Sangon Biotech. (Shanghai, China).

**Cellular fractionation, Co-immunoprecipitation (Co-IP), western blot and enzyme-linked sorbent assay (ELISA)**

To isolate cellular membrane, cytoplasm and nuclei components, cellular fractionation was performed according to manufacturer’s instruction. For Co-IP, primary antibodies at an immunoprecipitation level as well as 40 μl of protein G agarose beads were used to precipitate target proteins. The beads were washed in three rounds of lysis buffer and boiled in 50 μl of SDS loading buffer; the precipitates were then loaded on an SDS-PAGE gel processing for western blotting. Input lysates without any antibody precipitation and a rabbit normal IgG-precipitated samples were synchronically loaded for technical control.

For western blotting analysis, an equal amount of proteins was resolved by an 8-12% gel, transferred to polyvinylidene fluoride (PVDF) membranes (Millipore, Burlington, MA, USA), and incubated with appropriate primary antibodies at 4℃ overnight. Membranes were washed three times by Tris-buffered saline containing 0.1% tween-80 (TBST) and incubated with peroxidase-conjugated secondary antibodies. Protein bands were visualized with the enhanced chemiluminescence (ECL) reagent (Thermo Scientific Fisher) using a ChemiDoc MP Imaging System (Bio-Rad Co., Hercules, CA, USA).

To detect pyroptosis-associated cytokines, serum samples from animal experiments or supernatants from cell culture were subject to detection of IL‐1β and IL-18 using commercial ELISA kits in accordance with manufacturers’ instructions.

**Immunofluorescence assay, confocal microscopy, time-lapse microscopy and immunohistochemistry (IHC) staining**

Cultured cardiomyocytes were fixed in 4% paraformaldehyde, permeabilized in 0.1% Triton X-100 for 10 min, and blocked in 1× PBS supplemented with 5% normal bovine serum for 1h. Primary antibodies were incubated in blocking buffer at 4℃ overnight. Heart slices were also blocked in 5% normal bovine serum before incubation with primary antibodies. Secondary Alexa antibodies from Invitrogen were added for 1 h. Nuclei were counterstained with DAPI. Samples were then imaged under a Leica SP5 confocal microscope. For time-lapse microscopy, H9c2 myocytes were plated onto a 35-mm glass bottom dish (Cellvis, Mountain View, CA, USA) and were treated with Olz or Clz in complete DMEM containing 30 μM propidium iodide (PI) and imaged using a Leica SP8 confocal microscope under a 20× objective within an environmental chamber maintained at 37 °C and 5% CO2. For IHC staining, dewaxed slides were subjected to antigen retrieval in a microwave at 100°C for 10 min in 0.1 M citric acid buffer (PH 6.0), and were then incubated with primary antibodies at 4°C overnight. After secondary HRP-conjugated antibody incubation for 1 h, the immunoreactivity was developed in 0.05% diaminobenzidine containing 0.01% hydrogen peroxidase.

**Scanning electron microscopy (SEM) and transmission electron microscopy (TEM)**

After indicated treatments, H9c2 myocytes were fixed with 2.5% glutaraldehyde overnight, and then rinsed with PBS three times. The samples were dehydrated through a graded series of ethanol (50, 70, 90 and 100%) and dried by tertiary butanol method. Dried specimens were sputter coated with gold-palladium and imaged with a SU8010 cold field emission scanning electron microscope operating at 10 kV (Hitachi, Tyoko, Japan). For the TEM, mouse HL-1 myocytes were free to grow. When cell confluence reached approximate 70%, the cells were trypsinized and precipitated by centrifuge at 1,000 rpm for 5 min. Cell precipitates were then immersed in 2% glutaraldehyde. After overnight ﬁxation, samples were postﬁxed in 2% osmium tetraoxide, dehydrated through graded concentrations of ethanol, infiltrated and embedded in Epon 618 at 60℃ for 48 h. Ultrathin sections were cut with a diamond knife, mounted on formvar-coated slot grids, and then stained with 1% uranyl acetate and lead citrate. The images were taken with a CM-120 model FEI/PHILIPS TEM (Philips Electron Optics B.V., Eindhoven, The Netherlands).

**Sample preparation and LC-MS/MS analysis**

Quantitative detection of major endocannabinoids in blood samples or cultured cells were performed in accordance with a published method.^4^ For the detection of antipsychotics and their major metabolites, the plasma and tissue samples were thawed at room temperature before processing. An aliquot of 20 μL plasma was pipetted into a 1.5 mL Eppendorf tube and 80 μL of methanol-acetonitrile (1:1, v/v) with SKF-525A at the concentration of 100 ng/mL was added to precipitate the proteins. An equal of 20 mg section of tissue was weighed and placed in bead-beater tubes. Then 100 μL of methanol-acetonitrile (1:1, v/v) with SKF-525A at the concentration of 100 ng/mL was added to homogenize tissue samples. Both of the contents were vortex mixed for 40s and were centrifuged at 14,000 rpm for 15 min. The supernatant was subsequently aspirated for LC-MS/MS analysis.

The LC-MS/MS analysis was performed on an Agilent 1260 UPLC (Agilent, USA) coupled to an Agilent 6490 Triple Quad mass spectrometer (Agilent, USA) with the electrospray ionization (ESI) source. An Agilent poroshell 120 SB-Aq column (2.1 × 150 mm; 2.7μm) was used for separation with a flow rate at 0.4 mL/min and column temperature of 30 ℃. The mass parameters were as follows: capillary voltage was 4000 V, nozzle voltage was 500 V, gas temperature was 250 ℃, gas flow was 12 L/min, nebulizer was 35 psi, sheath gas temperature was 300 ℃, sheath gas flow was 11 L/min. Multiple Reaction Monitoring (MRM) was used to monitor clozapine, olanzapine and SKF-525A (Internal standard) in the positive ion mode. Data acquisition and processing were performed on Agilent Mass hunter workstation software (Agilent, USA).

**Surface Plasmon Resonance (SPR)**

A purified human CB1R (hCB1R) protein that is close to its full-length (473 amino acids) and verified to have active ligand binding properties^5^ is a generous gift from Professor Zhijie Liu from the iHuman Institute, Shanghai Technology University, Shanghai, China. The hCB1R protein was dissolved in 10 mM sodium acetate buffer (pH 4.0) and then immobilized on a CM5 sensor chip (GE Healthcare, Pittsburgh, PA, USA) using a Biacore T200 (GE Healthcare). The remaining active sites in the test flow cell were blocked by 1 M ethanolamine. The immobilization level was set at approximate 14,000 Biacore Response Unit (RU). Serial dilutions of analytes (AEA, olanzapine, or clozapine) were prepared using HBS-EP+ running buffer composed of 10 mM HEPES pH 7.4, 150 mM NaCl, 0.05% (v/v) Surfactant P20, and 3 mM EDTA, and were then flowed through the chip surface. Regeneration was achieved by injection of 10 mM NaOH (pH 10) after each sample injection. The signal was monitored by subtracting reference flow cell from test flow cell. The resulting data were fit to a 1:1 binding model using Biacore Evaluation Software (GE Healthcare) and obtained from kinetic analysis.

**References**

1 Li, L. L. *et al.* Opposite effects of cannabinoid CB1 and CB2 receptors on antipsychotic clozapine-induced cardiotoxicity. *Br. J. Pharmacol*. **176**, 890-905 (2019).

2 Li, X. Q. *et al.* Quetiapine induces myocardial necroptotic cell death through bidirectional regulation of cannabinoid receptors. *Toxicol. Lett*. **313**, 77-90 (2019).

3 Ackers-Johnson, M. *et al.* A Simplified, Langendorff-Free Method for Concomitant Isolation of Viable Cardiac Myocytes and Nonmyocytes From the Adult Mouse Heart. *Circ. Res*. **119**, 909-920 (2016).

4 Dong, X. R. *et al.* Surrogate analyte-based quantification of main endocannabinoids in whole blood using liquid chromatography-tandem mass spectrometry. *Biomed. Chromatogr*. **33**, e4439 (2019).

5 Hua, T. *et al.* Crystal Structure of the Human Cannabinoid Receptor CB1. *Cell*. **167**, 750-762, (2016).

**Supplementary Tables**

**Supplementary Table 1. Clinical and autopsy information for collected human heart samples.**

| **Category** | **Non-antipsychotics user with cardiac deaths (n=10)** | **Non-antipsychotics user with non-cardiac deaths**  **(n=10)** | **Antipsychotics users with cardiac deaths**  **(n=18)** | **Antipsychotics users with Non-cardiac deaths**  **(n=10)** |
| --- | --- | --- | --- | --- |
| Age, years | 55.7±15.8 | 40.8±16.0 | 48.2±10.8 | 47.3±16.3 |
| Gender, Male% | 60% | 50% | 66.7% | 50% |
| Duration of drug use, years | N/A | N/A | 18.0±8.4 | 11.0±10.0 |
| Heart weight, g | 380.7±94.4 | 269.1±37.7 | 383.9±82.5 | 322.4±55.3 |
| LV thickness, cm | 1.33±0.25 | 1.24±0.09 | 1.29±0.26 | 1.18±0.16 |
| RV thickness, cm | 0.33±0.08 | 0.28±0.09 | 0.33±0.11 | 0.30±0.08 |
| Daily AP medication (monotherapy%) | N/A | N/A | 66.7% | 70.0% |
| No. of cases positive for AP drugs at death (%). | N/A | N/A | 13 (72.2%) | 6 (60.0%) |

AP, antipsychotics. LV, left ventricle. RV, right ventricle. N/A, not applicable.

**Supplementary Table 2. Key resources table**

| **Reagents** | **Application** | **Source** | **Identifier** |
| --- | --- | --- | --- |
| **Antibody** |  |  |  |
| CB1R | wb, IF, IHC | Cell signaling Technology | Cat#93815; RRID: AB_2756361) |
| NLRP3 | wb | Cell signaling Technology | Cat#15101; RRID: AB_2722591 |
| NLRP3 | IF | Novus Biologicals | Cat#NBP2-12446; RRID: AB_2750946 |
| ASC | wb | Cell signaling Technology | Cat#67824; RRID: AB_2799736 |
| Caspase 1 | wb | Cell signaling Technology | Cat#24232; RRID: AB_2890194 |
| Caspase 1 | wb, IF | Proteintech | Cat#22915-1-AP; RRID: AB_2876874 |
| Cleaved Caspase 1 (p20) | wb | Cell signaling Technology | Cat#89332 |
| GSDMD | wb, IF | Abcam | Cat#ab209845; RRID: AB_2783550 |
| GSDMD (N-terminal) | wb, IHC | Affinity Biosciences | Cat# AF4013; RRID: AB_2846780 |
| IL-1β | wb | Cell signaling Technology | Cat#31202; RRID: AB_2799001 |
| mature IL-1β | wb | Wanleibio | Cat#WL00891; RRID: AB_2811279 |
| IL-18 | wb | Abcam | Cat#ab243091; RRID: AB_2861283 |
| GAPDH | wb | Cell signaling Technology | Cat#5174; RRID: AB_10622025 |
| β-Tubulin | wb | Cell signaling Technology | Cat#2128; RRID: AB_823664 |
| α-Actinin | wb | Proteintech | Cat#11313-2-AP; RRID: AB_2223815 |
| β-Actin | wb | Santa Cruz Biotechnology | Cat#sc-47778; RRID: AB_626632 |
| H-RAS | IF | Abcam | Cat#ab16907; RRID: AB_443543 |
| ANF | wb | Proteintech | Cat#27426-1-AP; RRID: AB_2880868 |
| MYH7 | wb | Proteintech | Cat#22280-1-AP; RRID: AB_2736821 |
| LC-3B | wb | Abcam | Cat#ab192890; RRID: AB_2827794 |
| Cardiac Troponin T | IF | Abcam | Cat#ab8295; RRID: AB_306445 |
| PARP | wb | Cell signaling Technology | Cat#9532; RRID: AB_659884 |
| Caspase 3 | wb | Cell signaling Technology | Cat#14220; RRID: AB_2798429 |
| BAX | wb | Cell signaling Technology | Cat#2772; RRID: AB_10695870 |
| RIPK3 | wb | Proteintech | Cat#17563-1-AP; RRID: AB_2178659 |
| MLKL | wb | Proteintech | Cat#66675-1-Ig; RRID: AB_2882029 |
| Beclin 1 | wb | Proteintech | Cat#11306-1-AP; RRID: AB_2259061 |
| ATP1α1 | wb | Proteintech | Cat#14418-1-AP; RRID: AB_2227873 |
| Lamin B1 | wb | Proteintech | Cat#12987-1-AP; RRID: AB_2136290 |
| Anti-Flag tag Rabbit antibody | IP | Proteintech | Cat#20543-1-AP; RRID: AB_11232216 |
| Anti-Flag tag mouse antibody | IP | Affinity Biosciences | Cat#T0003; RRID: AB_2839412 |
| Anti-Myc tag antibody | IP | Proteintech | Cat#16286-1-AP; RRID: AB_11182162 |
| Anti-His tag antibody | IP | Proteintech | Cat#66005-1-Ig; RRID: AB_11232599 |
| Anti-HA tag antibody | IP | Proteintech | Cat#51064-2-AP; RRID: AB_11042321 |
| Anti-GST tag antibody | IP | Proteintech | Cat#66001-2-Ig; RRID: AB_2881488 |
| HRP-conjugate goat anti-mouse IgG (H+L) | Wb, IHC | Jackson ImmunoResearch Lab. | Cat#115-035-166; RRID：AB_2338511 |
| HRP-conjugate goat anti-rabbit IgG (H+L) | Wb, IHC | Jackson ImmunoResearch Lab. | Cat#111-035-144; RRID: AB_2307391 |
| Goat anti-rabbit secondary antibody Alexa Fluor 555 | IF | Invitrogen | Cat# A-21428; RRID: AB_2535849 |
| Goat anti-rabbit Alexa Fluor 488 | IF | Invitrogen | Cat#A-11008; RRID: AB_143165 |
| **Chemicals** |  |  |  |
| Olanzapine |  | Sellckchem | Cat#2493 |
| Clozapine |  | Sellckchem | Cat#S2459 |
| Quetiapine |  | Selleckchem | Cat#S1763 |
| SKF-525A |  | MedChem Express | Cat#HY-B1311 |
| Rimonabant (SR141716) |  | Selleckchem | Cat#S3021 |
| AM 251 |  | MedChem Express | Cat#HY-15443 |
| AM 281 |  | APExBio Technology | Cat#B6603 |
| ACEA |  | Tocris Bioscience | Cat#1319 |
| MCC950 (CP-456773) |  | Selleckchem | Cat#S7809 |
| VX-765 (Belnacasan) |  | MedChem Express | Cat#HY-13205 |
| Apoptosis inhibitor I/II |  | Chemical Library of CAS | N/A |
| 3-methyladenine |  | MedChem Express | Cat# HY-19312 |
| wortamanin |  | Selleckchem | Cat# S2758 |
| Necrostatin-1 |  | Santa Cruz Biotechnology | Cat# 4311-88-0 |
| Polybrene |  | Sigma-Aldrich | Cat#TR-1003 |
| Bovine Serum Albumin |  | Beyotime Biotechnology | Cat#ST-023 |
| Dulbecco's modified eagle medium |  | Gibco | Cat#11995065 |
| Fetal Bovine Serum |  | Gibco | Cat# 10099141 |
| Penicillin-streptomycin |  | Gibco | Cat#15140-122 |
| CM5 sensor chip |  | GE Healthcare | Cat#29104988 |
| **Critical commercial assays** |  |  |  |
| Mouse IL-1β ELISA kit |  | Jiancheng Bioengineering | Cat#H-002 |
| Mouse IL-18 ELISA kit |  | Jiancheng Bioengineering | Cat#H-015 |
| Lipofectamine^TM^ 3000 Reagent |  | Invitrogen | Cat#L3000001 |
| Membrane, nuclear and cytoplasmic protein extraction kit |  | Sangon Biotech | Cat# C510002 |
| Serum Triglycerides Assay Kit |  | Jiancheng Bioengineering | Cat#F001-1-1 |
| Serum Total Cholesterol Assay Kit |  | Jiancheng Bioengineering | Cat#F002-1-1 |
| Creatinine Assay kit |  | Jiancheng Bioengineering | Cat# C011-2-1 |
| Urea Assay Kit |  | Jiancheng Bioengineering | Cat# C013-2-1 |
| Aspartate aminotransferase (AST) Kit |  | Jiancheng Bioengineering | Cat# C010-2-1 |
| Alanine aminotransferase (ALT) Kit |  | Jiancheng Bioengineering | Cat# C009-2-1 |
| Albumin assay kit |  | Jiancheng Bioengineering | Cat# A028-2-1 |
| Glucose Assay Kit |  | Jiancheng Bioengineering | Cat# F006-1-1 |
| HiScript II Q RT SuperMix |  | Vazyne | Cat#R223 |
| AceQ qPCR SYBR Green Master Mix |  | Vazyme | Cat#Q121 |
| **Experimental Models: cell lines** |  |  |  |
| Mouse HL-1 cells |  | Millipore | Cat# SCC065; RRID: CVCL_0303 |
| Rat H9c2 cells |  | ATCC | ATCC® CRL-1446; RRID: CVCL_0286 |
| Human AC-16 cells |  | Millipore | Millipore Cat# SCC109; RRID: CVCL_4U18 |
| **Experimental Models: organisms/strains** |  |  |  |
| C57BL/6J WT |  | Shanghai Laboratory Animal Center | RRID: IMSR_JAX:000664 |
| Balb/C WT |  | Shanghai Laboratory Animal Center | MGI: 6272006 |
| *Cb1r*^-/-^ |  | Shanghai Model Organisms | N/A |
| *Nlrp3^-/-^* |  | In house breeding | N/A |
| *Gsdmd^-/-^* |  | Provided by Prof. Feng Shao | N/A |
| **Software and Algorithms** |  |  |  |
| Prism 8.0 |  | GraphPad software Inc. | N/A |
| Excel 16.0 |  | Microsoft | N/A |
| **Recombinant DNA** |  |  |  |
| Flag-CB1R | Co-IP | This paper | N/A |
| GST-ASC | Co-IP | This paper | N/A |
| Myc-NLRP3 | Co-IP | This paper | N/A |
| His-GSDMD | Co-IP | This paper | N/A |
| HA-Casp1 | Co-IP | This paper | N/A |
| **Oligonucleotides (5’-3’)** |  |  |  |
| *mNlrp1b-F:* GGTGGTGTGAAGATGTTGTGT | qPCR | This paper | N/A |
| *mNlrp1b-R:* TCCATGTTCATCGTAGGGACC | qPCR | This paper | N/A |
| *mNlrp3-F:* ATTACCCGCCCGAGAAAGG | qPCR | This paper | N/A |
| *mNlrp3-R:* TCGCAGCAAAGATCCACACAG | qPCR | This paper | N/A |
| *mCasp1-F:* ACAAGGCACGGGACCTATG | qPCR | This paper | N/A |
| *mCasp1-R:* TCCCAGTCAGTCCTGGAAATG | qPCR | This paper | N/A |
| *mAsc-F:* CTTGTCAGGGGATGAACTCAAAA | qPCR | This paper | N/A |
| *mAsc-R:* GCCATACGACTCCAGATAGTAGC | qPCR | This paper | N/A |
| *mGsdmd-F:* CCATCGGCCTTTGAGAAAGTG | qPCR | This paper | N/A |
| *mGsdmd-R:* ACACATGAATAACGGGGTTTCC | qPCR | This paper | N/A |
| *mCb1r-F:* AAGTCGATCTTAGACGGCCTT | qPCR | This paper | N/A |
| *mCb1r-R:* TCCTAATTTGGATGCCATGTCTC | qPCR | This paper | N/A |
| *mGapdh-F:* AGGTCGGTGTGAACGGATTTG | qPCR | This paper | N/A |
| *mGapdh-R:* TGTAGACCATGTAGTTGAGGTCA | qPCR | This paper | N/A |
| m18S rRNA-F: GGGAGCCTGAGAAACGGC | qPCR | This paper | N/A |
| m18S rRNA-R: GGGTCGGGAGTGGGTAATTT | qPCR | This paper | N/A |
| *shCb1r:* TGAAGGATGACACATAGCACC |  | This paper | N/A |
| *shNlrp3:* TCTAGTGTTCTTGCTGACTGC |  | This paper | N/A |
| *shNlrp1b:* AGAAGAAGACATGCTGGAAGC |  | This paper | N/A |
| *shCasp1:* CTATATGGGCCTTCTTAATGC |  | This paper | N/A |
| *shGsdmd:* TATACACACATTCATGGAGGC |  | This paper | N/A |
| *Cb1r*-KO guide sequence 1: tatctgcaaggccgtctaag | CRISPR-guided *Cb1r* KO | This paper | N/A |
| *Cb1r*-KO guide sequence 2: aacgaggacaacatccagtg | CRISPR-guided *Cb1r* KO | This paper | N/A |
| *Cb1r*-KO guide sequence 3: gagcactgttaagatcgcca | CRISPR-guided *Cb1r* KO | This paper | N/A |
| *Cb1r*-KO guide sequence 4: ctgtgttattggcgtgcttg | CRISPR-guided *Cb1r* KO | This paper | N/A |
| Flag-*Cb1r*-C416S-F: TGTTCCCTTCAgcTGAAGGCACTGCGCAGCCTC | Point mutants | This paper | N/A |
| Flag-*Cb1r*-C416S-R: CTTCAgcTGAAGGGAACATGCTGCGGAAAGCAT | Point mutants | This paper | N/A |
| Flag-*Cb1r*-S426S-F: TCTAGATAACgcCATGGGGGACTCAGACTGCCT | Point mutants | This paper | N/A |
| Flag-*Cb1r*-S426S-R: CCCATGgcGTTATCTAGAGGCTGCGCAGTGCCT | Point mutants | This paper | N/A |
| Flag-*Cb1r*-S430S-F: TAACAGCATGGGGGACgCAGACTGCCTGCACAAGCACG | Point mutants | This paper | N/A |
| Flag-*Cb1r*-S430S-R: cGTCCCCCATGCTGTTATCTAGAGGCTGCGCA | Point mutants | This paper | N/A |
| Flag-*Cb1r* M1-F: CCGCTCGAGggctcaaatgacattcagta | Deletion mutants | This paper | N/A |
| Flag-*Cb1r* M2-F: CCGCTCGAGcttcactcccgcagtctccg | Deletion mutants | This paper | N/A |
| Flag-*Cb1r* M3-F: CCGCTCGAGgacttccacgtgttccaccg | Deletion mutants | This paper | N/A |
| Flag-*Cb1r* M4-F: CCGCTCGAGctcacggccatcgacaggta | Deletion mutants | This paper | N/A |
| Flag-*Cb1r* M5-F: CCGCTCGAGggctggaactgcaagaagct | Deletion mutants | This paper | N/A |
| Flag-*Cb1r* M6-F: CCGCTCGAGtggaaggctcacagccacgc | Deletion mutants | This paper | N/A |
| Flag-*Cb1r* M7-F: CCGCTCGAGgatgtctttgggaagatgaa | Deletion mutants | This paper | N/A |
| Flag-*Cb1r* M8-F: CCGCTCGAGctgaggagcaaggacctgag | Deletion mutants | This paper | N/A |
| Flag-*Cb1r* deletion Mutants-R: CGCGGATCCTCACTTATCGTCGTCATCCTT | Deletion mutants | This paper | N/A |

**Supplementary Figure Legends**

**
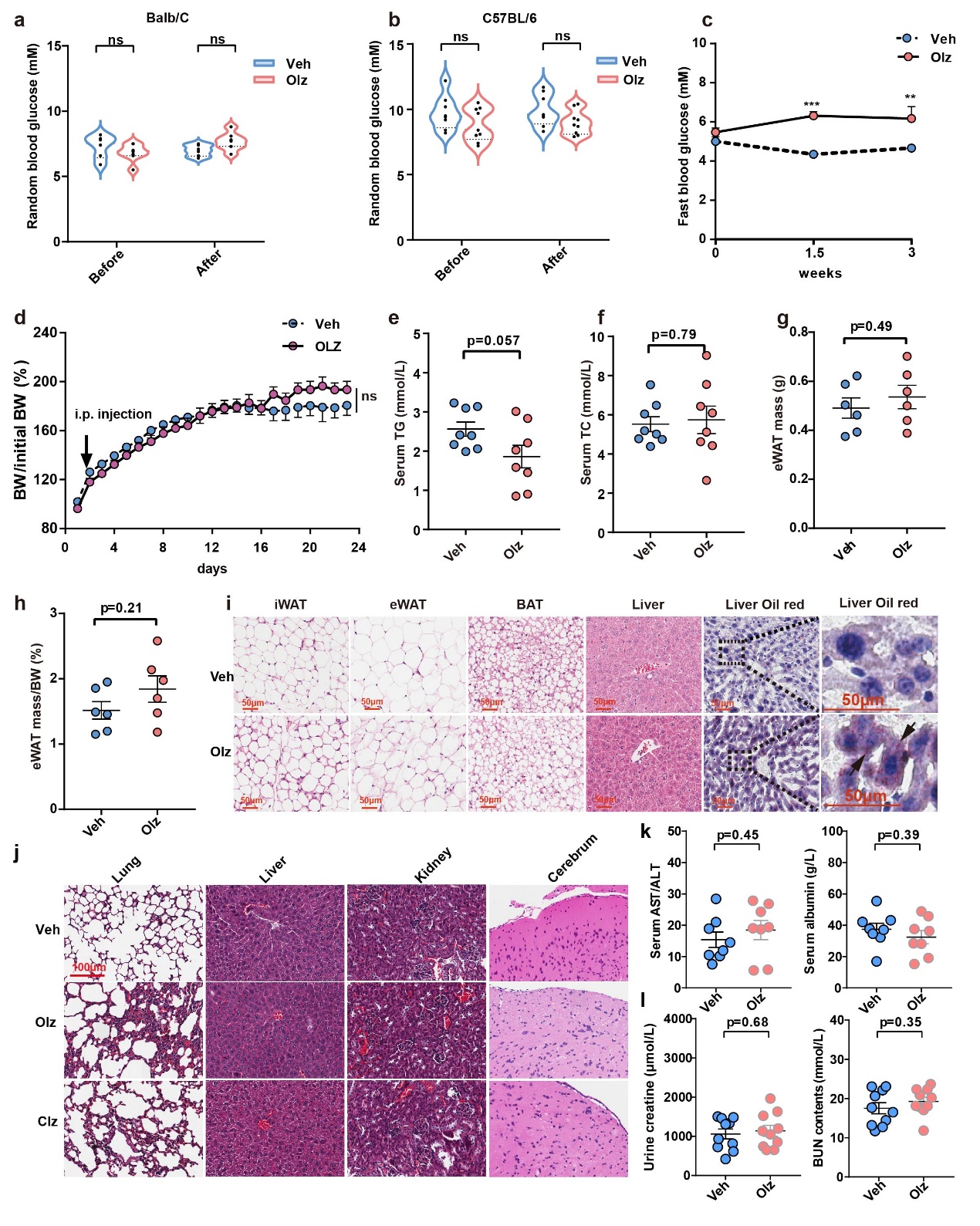
**

**Supplementary Fig. 1 Olanzapine (Olz) caused cardiotoxicity before inducing glycolipid metabolism disorder and other organ dysfunctions**. **a**, Random blood glucose levels of Balb/C mice before and after 21-day Olz treatments (5mg/kg, n=5-6/group). **b**, Random blood glucose levels of C57BL/6 mice before and after 21-day Olz treatments (5mg/kg, n=5-6/group). **c**, Fast blood glucose levels of C57BL/6 mice before Olz treatments (week 0), after 1.5-week Olz treatments (week 1.5), and after 3-week Olz treatments (week 3) (n=5-6/group). **d**, The percentage of body weight (BW) to initial BW were shown over the treatment days (n=5-6/group). **e, f**, Total triglycerides (TG) and total cholesterol (TC) levels were detected with biochemical assays (n=8/group). **g, h**, Epididymal white adipose tissue (eWAT) mass and the percentage of eWAT to BW were calculated and shown (n=6/group). **i**, H&E staining of major glycolipid metabolic organs including inguinal white adipose tissue (iWAT), eWAT, brown adipose tissue (BAT), and liver tissues. The livers were also stained with Oil red to identify any lipid droplet accumulation. Black arrows indicated a halo of lipid droplet. **j**, H&E staining of lung, liver, kidney and cerebrum in Veh (PBS), Olz (5mg/kg), and clozapine (Clz, 25mg/kg)-treated mice. Representative images were shown. **k, l**, Biochemical detection of liver function markers (serum AST, ALT, and albumin) and kidney function markers (urine creatine, blood urea nitrogen) in Veh- and Olz-treated mice (n=8-10 mice/group). Scale bar=100 μm or 50 μm as indicated. Data were expressed as mean ± SEM. The Student’s *t*-test was used for analysis in (**a, b, e-h, l**). Two-way ANOVA was used for analysis in (**c, d**). Veh, vehicle (PBS). ns, no significance. Other *p* values were as indicated.

**
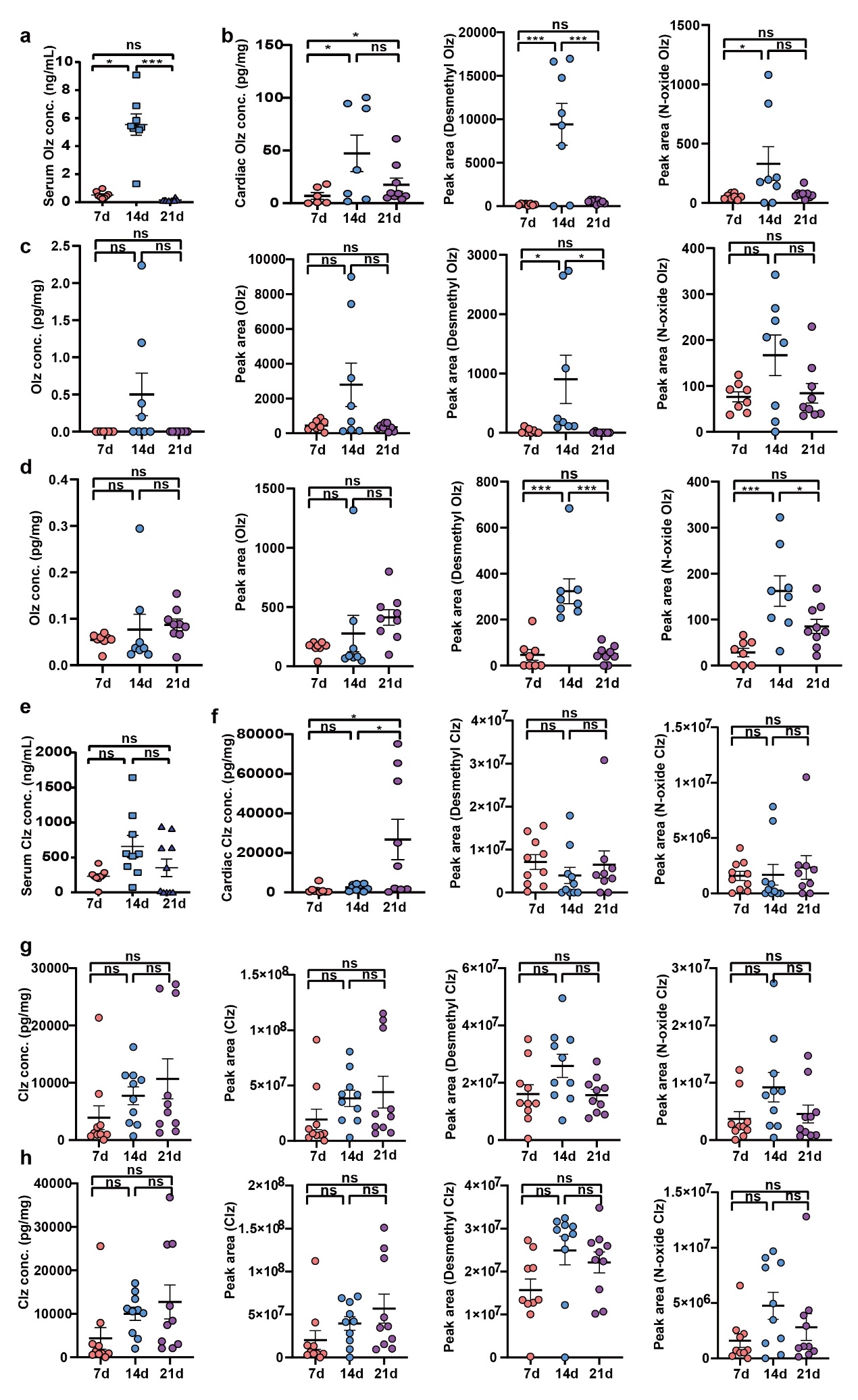
**

**Supplementary Fig. 2 Olanzapine (Olz) and Clozapine (Clz) accumulated specifically in mouse hearts within 21 days (n=8-10 mice/group).** Olz (5mg/kg) and Clz (25mg/kg) were intraperitoneally injected into mice. Serum, heart, liver, and kidney samples were collected immediately after mice sacrifice. **a**, Serum Olz concentrations after 7d, 14d, and 21d treatments. **b**, Olz concentrations (pg/mg) and its major metabolites (dimethyl Olz and N-oxide Olz, intensity expressed as peak area) in the heart. **c**, The Olz concentration (pg/mg) and the peak areas of Olz and its metabolites were detected in the liver. **d**, The Olz concentration (pg/mg) and the peak areas of Olz and its metabolites were detected in the kidney. **e**, Serum Clz concentrations after 7d, 14d, and 21d treatments. **f**, Clz concentrations (pg/mg) and its major metabolites (dimethyl Clz and N-oxide Clz, intensity expressed as peak area) in the heart. **g**, The Clz concentration (pg/mg) and the peak areas of Clz and its metabolites were detected in the liver. **h**, The Clz concentration (pg/mg) and the peak areas of Clz and its metabolites were detected in the kidney. Data were expressed as mean ± SEM. One-way ANOVA with Bonferroni post-hoc test was used for all analyses. ns, no significance. **p*<0.05; ****p*<0.001 as indicated.

**
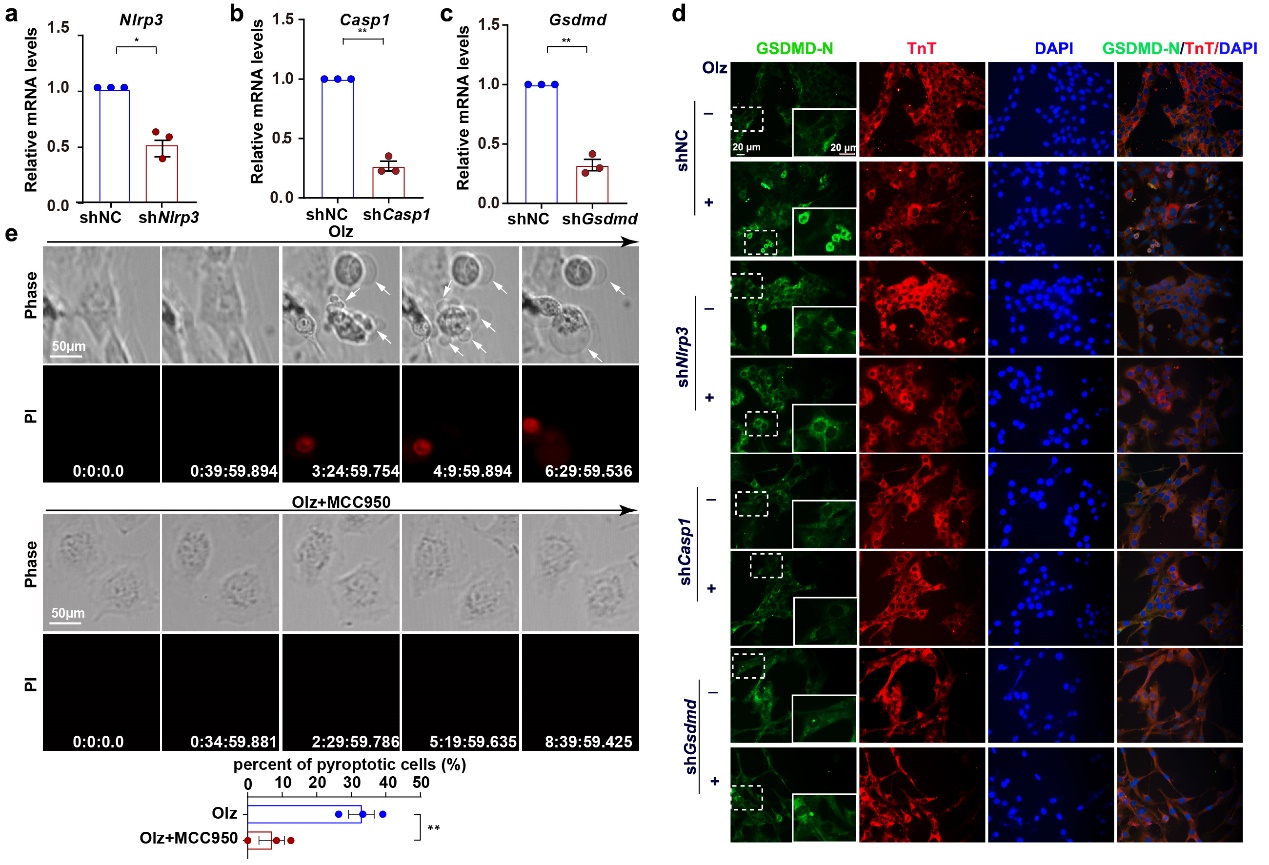
**

**Supplementary Fig. 3 Olanzapine (Olz) induced cell pyroptosis dependent of the NLRP3 inflammasome**. **a-c**, A pool of shRNAs targeting the major genes of cell pyroptosis were designed and their efficiency were confirmed by qRT-PCR in three independent assays in cardiac HL-1 cells. **d**, Effects of each gene knockdown on Olz (4 μM)-induced GSDMD membrane gathering were assessed using immunofluorescence assay in cardiac HL-1 cells. Insets at lower right corners represents local magnification from the GSDMD staining images. Scale bar=20 μM. White arrows indicated GSDMD membrane-positive cells. **e**, Rat H9c2 cells were pretreated with MCC950 (1 μM) for 1 hour. The cells were then subject to Olz treatment (4 μM) in medium containing PI dye (30 μM). White arrows indicated pyroptotic protrusions. Scale bar=50 μm. The percent of pyroptotic cells (defined as cells with pyroptotic protrusions and PI uptake) was quantified from three independent assays. Student’s *t*-test was used for statistical analysis in (a, b, c, and e). **p*<0.05; ***p*<0.01 as indicated.

**
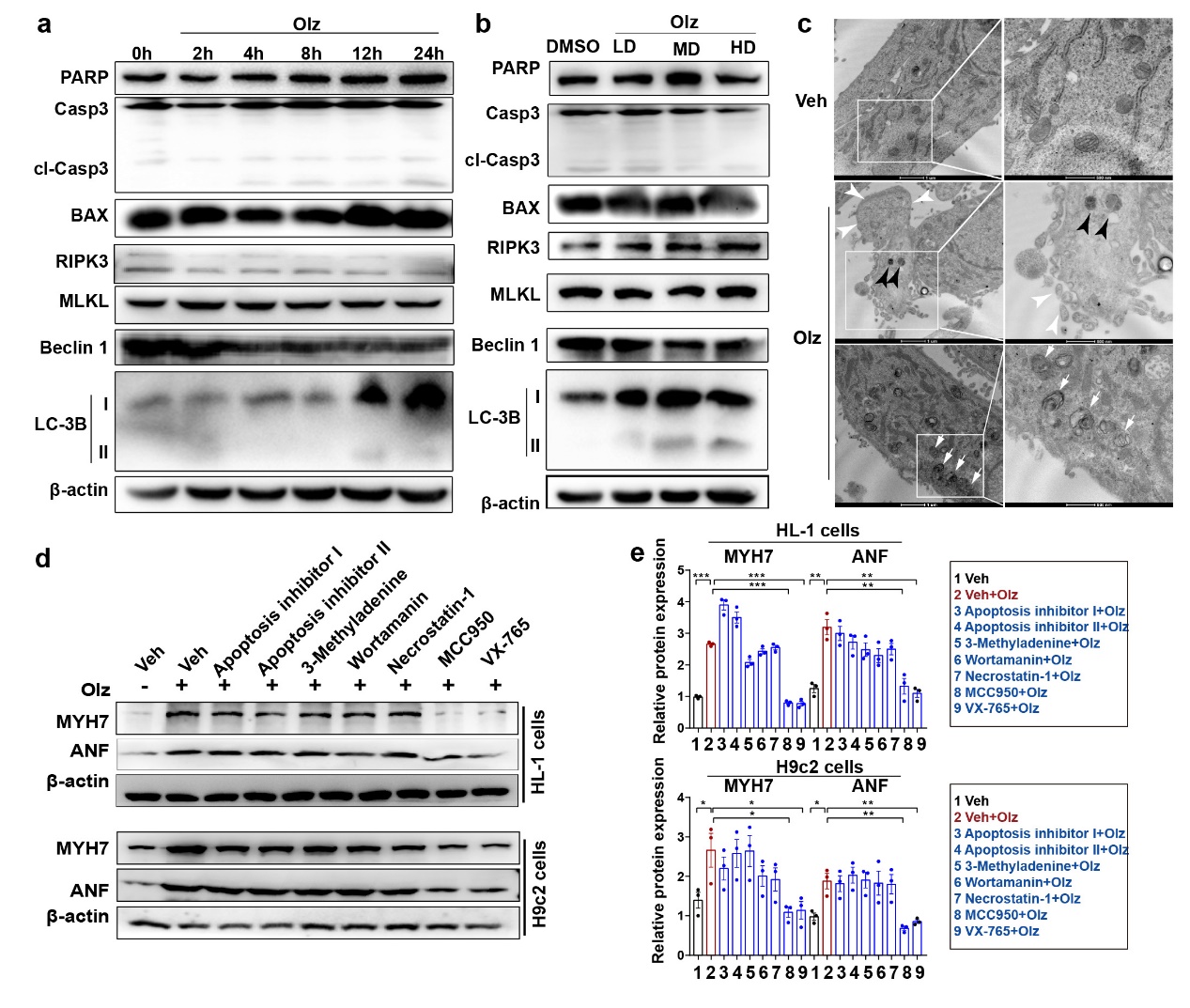
**

**Supplementary Fig. 4 Olanzapine (Olz) had minimal effects on other types of programmed cell death**. **a, b**, Western blot analysis of apoptosis markers (PARP, caspase 3, cleaved caspase 3, and BAX), necroptosis markers (RIPK3, MLKL), and autophagy markers (Beclin 1, LC3B) in mouse HL-1 cells under Olz treatments for different hours or doses. LD, low dose (1 μM). MD, medium dose (4 μM). HD, high dose (16 μM). **c**, Transmission electron microscope observation of HL-1 cells with or without Olz (8 μM) treatments. Olz-treated cardiac cells showed disintegrity of cell membrane and organelle swelling and increased number of autolysosomes. White arrowhead indicated membrane protrusion and black arrowheads indicated vesicles containing electron-dense content. White arrows indicate autophageosome/autolysosome. Scale bar=1 μm for left images and 500 nm for right images. **d, e**, Specific pharmacologic inhibitors of apoptosis (apoptosis inhibitor I and II, 10 μM), autophagy (3-methyladenine, 40 μM and wortamanin, 1 μM), necroptosis (necrostatin-1, 60 μM) and pyroptosis (MCC950, 10 nM and VX-765, 2 μM) were co-treated with Olz (4 μM). Cell proteins were then extracted and subject to western blot analysis of myocardial injury markers (MYH7, ANF) in mouse HL-1 myocytes and rat H9c2 myocytes. The protein intensity was then normalized to β-actin in three independent assays. Veh, vehicle (PBS). *, *p*<0.05; **, *p*<0.01; ***, *p*<0.001 as indicated.

**
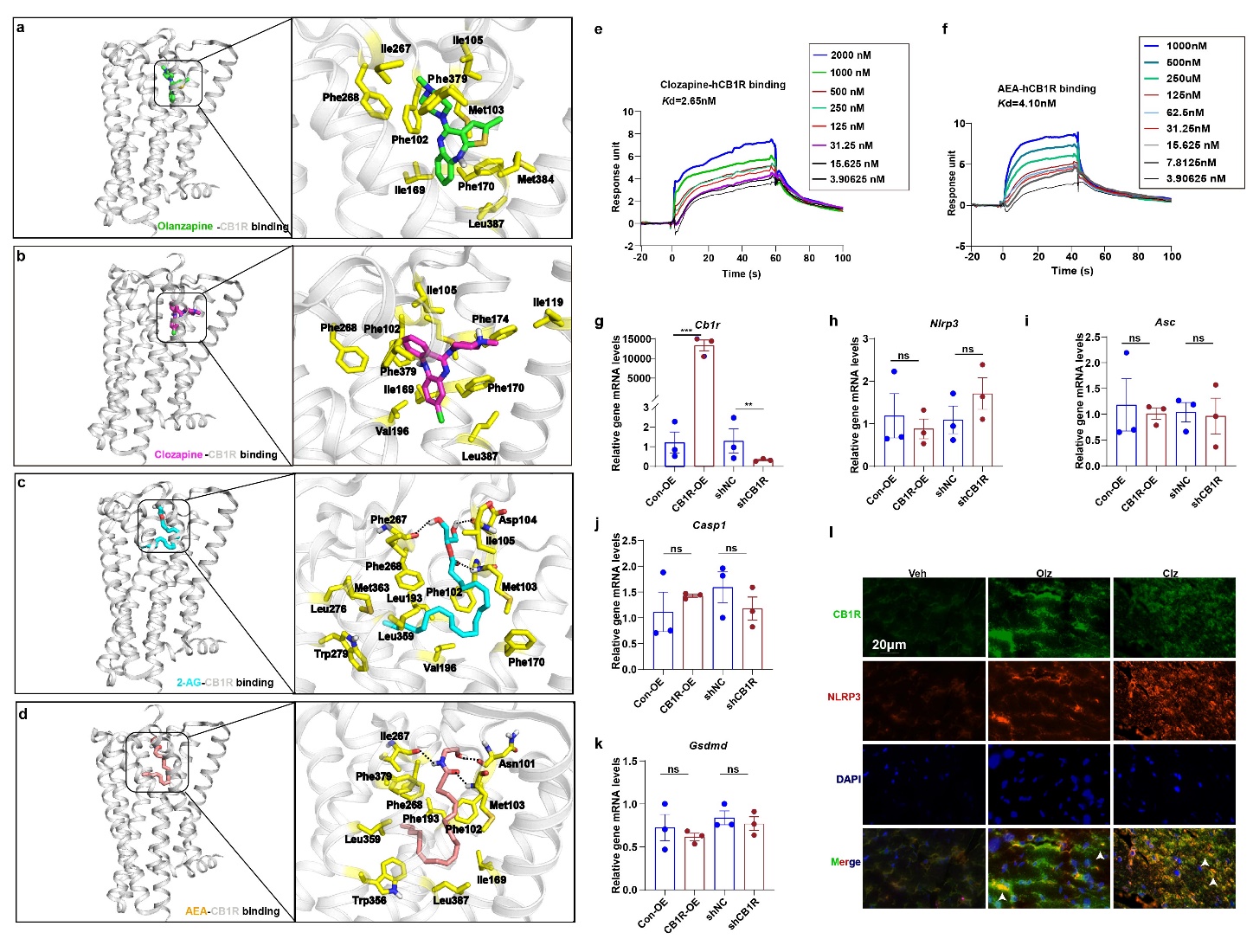
**

**Supplementary Fig. 5 Antipsychotics competed with endogenous ligands in binding to hCB1R and induced CB1R co-localization with NLRP3 in mouse hearts**. **a, b**, The conformation of AP drugs (Olz in green stick and Clz in purple stick) binding with human CB1R (hCB1R, grey cartoon). **c, d**, The conformation of major endocannabinoids (2-AG in cyan stick and AEA in orange stick) binding with hCB1R (grey cartoon). Right panels show the magnified view of the binding pockets in hCB1R. Key CB1R residues (yellow stick) involved in ligand binding are shown in stick representation. **e, f**, Surface plasmon resonance (SPR) measurements illustrating binding of Clz or AEA binding to the hCB1R. **g-k**, HL-1 myocytes were transiently transfected with a recombined *Cb1r* overexpression (CB1R-OE) plasmid, or a short-hairpin RNA-delivered silencing plasmid of *Cb1r* (shCB1R). A vector control plasmid (Con-OE) or a negative control shRNA (shNC) was respectively transfected as the corresponding controls. qRT-PCR analysis of *Cb1r*, *Nlrp3*, *Asc*, *Casp1*, and *Gsdmd* in three independent assays. **l**, Immunofluorescence analysis of CB1R (green signal) and NLRP3 (red signal) in Veh (PBS), Olz (5 mg/kg), and Olz (25 mg/kg)-treated mouse hearts. Arrowheads indicated co-localization of CB1R and NLRP3. Data were expressed as mean ± SEM. Unpaired Student’s *t*-test was used for all analyses. ***p*<0.01; ****p*<0.001 as indicated. ns, not significance.

**
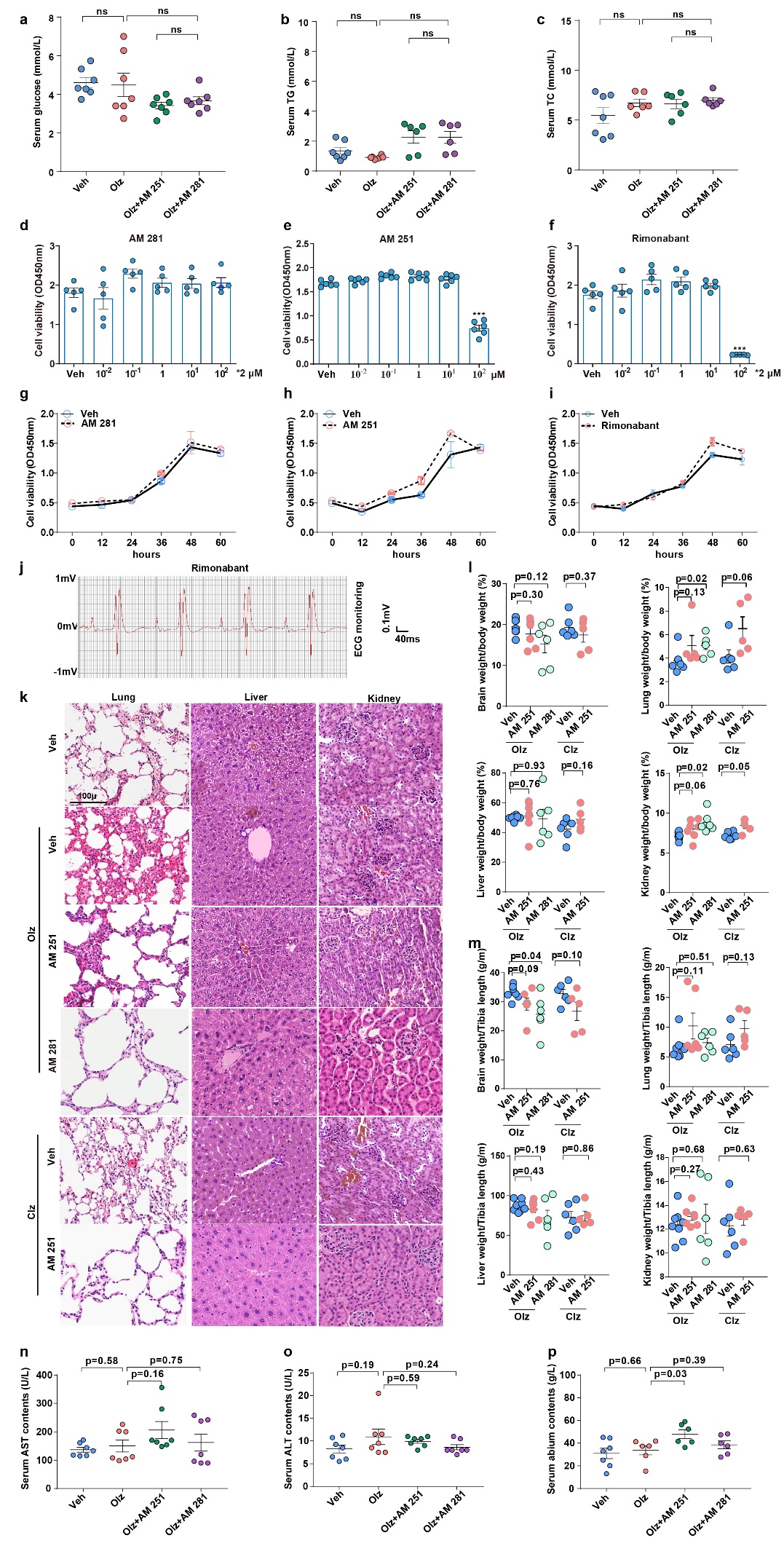
**

**Supplementary Fig. 6 CB1R antagonists AM 251 and AM 281 did not cause additional metabolic side effects or toxic effects to solid organs**. **a-c**, Serum levels of glucose, total triglycerides (TG), and total cholesterol (TC) were detected using biochemical assays (n=7 mice/group). **d-f**, Three CB1R antagonists (AM 251, AM 281, and Rimonabant) were treated with HL-1 cells at gradient doses for 24 hours. Cell counting kit-8 (CCK-8) assay was performed to detect cell viability (n=5-6 replicates/group). **g-i**, Three CB1R antagonists at final doses of 1 μM were treated with HL-1 cells for different hours. CCK-8 assay was performed to detect cell viability (n=3/group). **j**, Electrocardiography monitoring showed high amplitude of J-wave caused by Rimonabant. **k**, H&E staining of lung, liver and kidney tissues under indicated treatments. **l**, Ratios of brain weight, lung weight, liver weight, and kidney weight to body weight were shown for the indicated groups (n=6-8 mice/group). **m**, Ratios of brain weight, lung weight, liver weight, and kidney weight to right tibia length were shown for the indicated groups (n=6-8 mice/group). **n-p**, Serum AST, ALT and albumin contents were detected using biochemical assays for indicated groups (n=7 mice/group). Data were expressed as mean ± SEM. One-way ANOVA was used for analysis in (**d-i**). The student’s *t*-test was used for analysis in (**a-c**, **l-p**). Veh, vehicle (PBS). ****p*<0.001 *vs.* Veh or *p* values as marked. ns, no significance.

**Supplementary Videos**

**Supplementary video 1.** Real-time video of a representative field recorded immediately after stimulation of H9c2 cells with Olanzapine (Olz, 4 μM) (the exact time duration, h: min: s. ms). Scale bar=75 μm.

**Supplementary video 2.** Real-time video of a representative field recorded immediately after stimulation of H9c2 cells with Clozapine (Clz, 20 μM) (the exact time duration, h: min: s. ms). Scale bar=75 μm.
