# Supplementary figures and images for: CB1R-stabilized NLRP3 inflammasome drives antipsychotics cardiotoxicity
Source: Signal Transduct Target Ther. 2022 Jun 24;7:190. doi: 10.1038/s41392-022-01018-7 (PMC9225989; doi:10.1038/s41392-022-01018-7)

Figure 1H

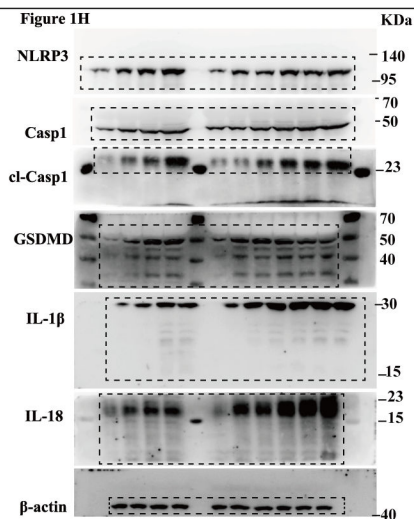

Figure 1I

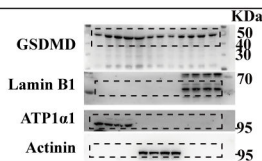

Figure 1M

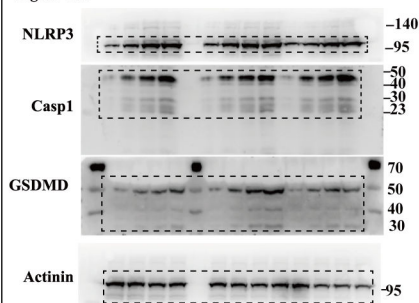

Figure 2F

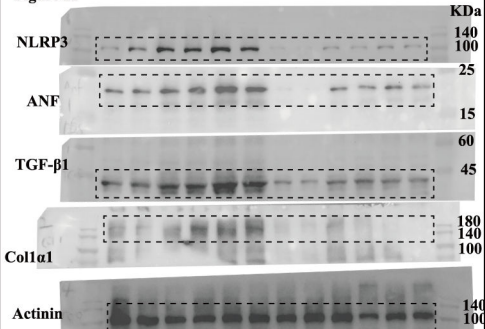

Figure 2N

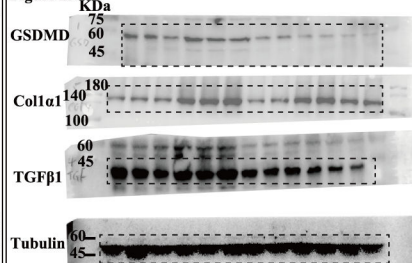

Figure 2J

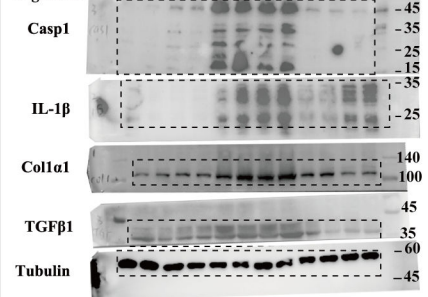

Figure 3C

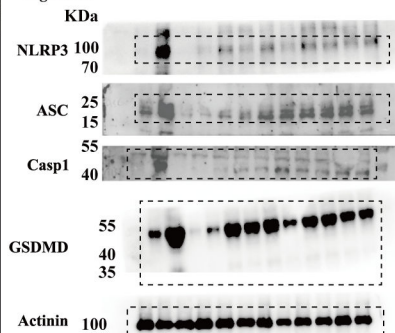

Figure 3H

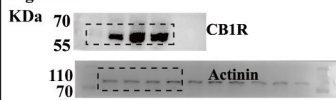

Figure 3L

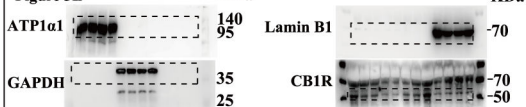

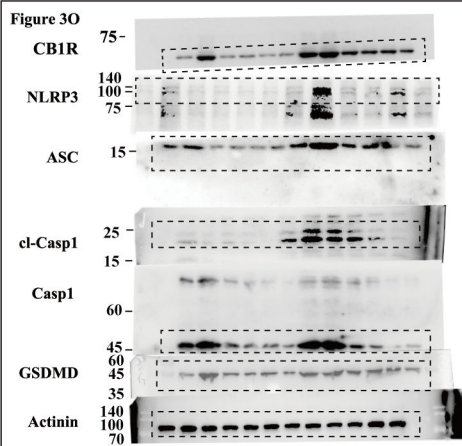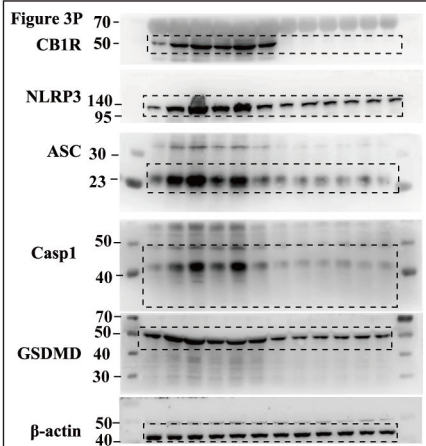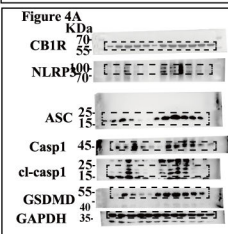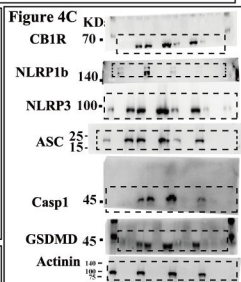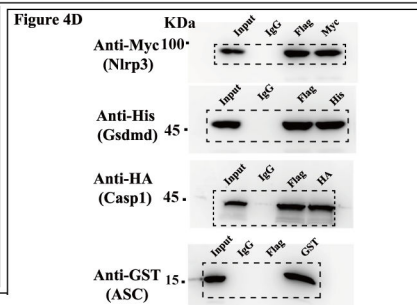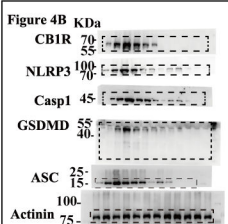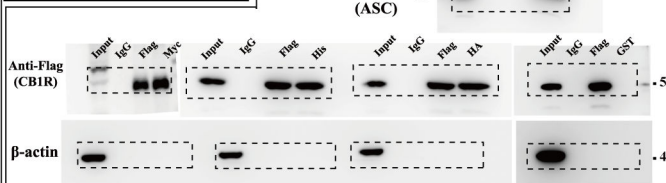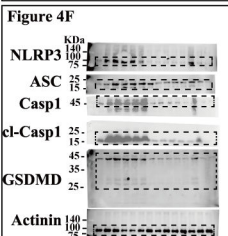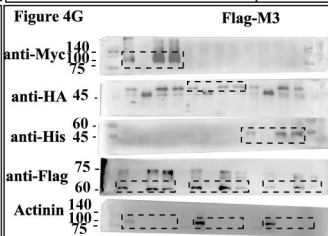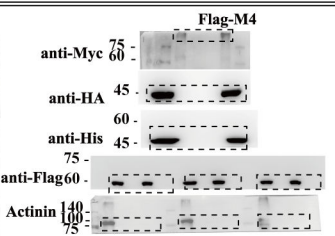

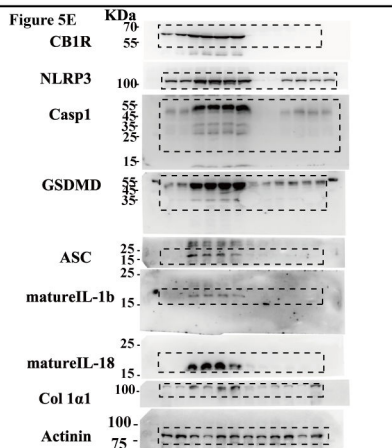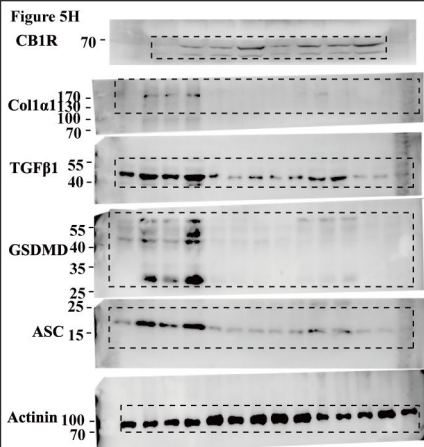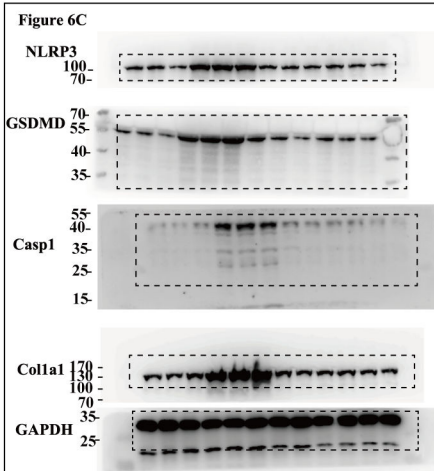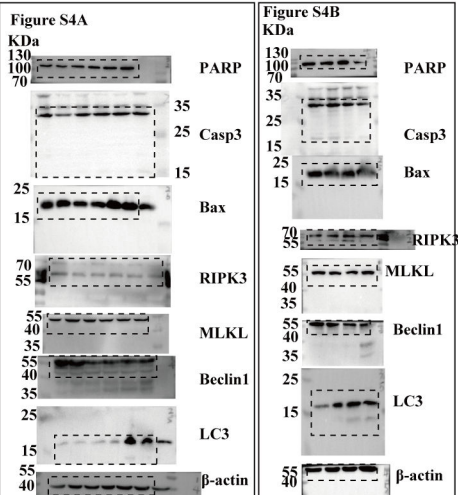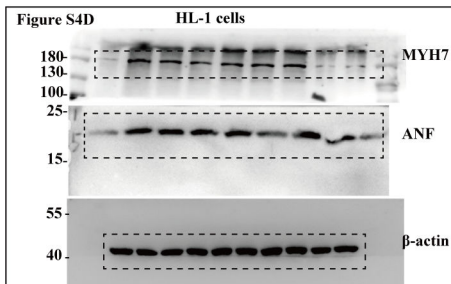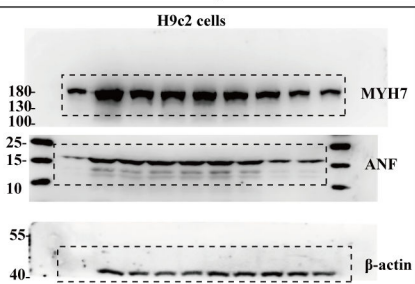

Supplement: Supplementary file 4 — Raw western blots [file 41392_2022_1018_MOESM4_ESM.pdf]
